# Hippocampal Astrocyte Morphology Follows an Unexpected Trajectory With Age in a Transgenic Rodent Model of Tauopathy

**DOI:** 10.1002/glia.70019

**Published:** 2025-03-22

**Authors:** Emma Augustin, Tatiana Vinasco‐Sandoval, Miriam Riquelme‐Perez, Damien Plassard, Mylène Gaudin, Gwenaëlle Aurégan, Julien Mitja, Sueva Bernier, Charlène Joséphine, Fanny Petit, Caroline Jan, Anne‐Sophie Hérard, Marie‐Claude Gaillard, Agathe Launay, Emilie Faivre, Luc Buée, Anne‐Laurence Boutillier, David Blum, Alexis‐Pierre Bemelmans, Gilles Bonvento, Karine Cambon

**Affiliations:** ^1^ Université Paris‐Saclay, CEA, CNRS, MIRCen Laboratoire Des Maladies Neurodegeneratives Fontenay‐aux‐Roses France; ^2^ CEA, CNRS, DRF, IBFJ, IRCM Laboratoire de Génomique et Radiobiologie de la Kératinopoeièse Evry France; ^3^ GenomEast Platform, Institut de Génétique et de Biologie Moléculaire et Cellulaire (IGBMC), CNRS UMR 7104, INSERM U1258 Université de Strasbourg Illkirch France; ^4^ Université Paris‐Saclay CNRS, Institut Des Neurosciences Paris‐Saclay Saclay France; ^5^ Université de Lille, Inserm, CHU Lille Lille France; ^6^ Alzheimer and Tauopathies LabEx DISTALZ Lille France; ^7^ Laboratoire de Neurosciences Cognitives et Adaptatives (LNCA) Université de Strasbourg Strasbourg France; ^8^ UMR 7364 Centre National de la Recherche Scientifique (CNRS) Strasbourg France

**Keywords:** aging, astrocytes, morphology, tau

## Abstract

Individual protoplasmic astrocytes have very complex and diverse spongiform shapes. The morphological diversity of astrocytes is determined by the structural and functional interactions of the astrocyte with its microenvironment. When faced with pathological conditions, astrocytes reorganize their morphology. Yet, little is known about the astrocytic response in pure tauopathies and its evolution over time. Here, we aimed to investigate the consequences of a primary neuronal tau pathology on astrocyte fine morphology at three stages of the disease using the transgenic Thy‐Tau22 mouse model. We first showed that hippocampal astrocytes in Thy‐Tau22 mice progressively accumulate hyperphosphorylated tau with age. We then developed a pipeline of analyses, including 3D reconstruction of hippocampal tdTomato‐labeled astrocytes via a PHP.eB adeno‐associated virus, confocal microscopy, Imaris software morphometric analysis, and an advanced statistical analysis. During normal aging, the complexity of astrocyte morphology peaked at adulthood, then declined. In contrast, in Thy‐Tau22 mice, tauopathy was associated with a simpler initial morphology, followed by the appearance of a cluster of complex cells at the most advanced stage. Using principal component analysis and hierarchical clustering based on 10 morphological features, we were able to identify different astrocyte morphotypes whose relative proportion varies differently with age between WT and Thy‐Tau22 mice. Interestingly, we revealed that a fraction of astrocytes with a complex morphology re‐emerges late in tauopathy‐affected animals. Our data highlight the concept of significant and reversible structural plasticity of astrocytes when faced with chronic pathological conditions.

## Main Points

- During ageing, the complexity of astrocyte morphology peaks at adulthood then declines.
- In Tau22 transgenic mice, tau accumulates in astrocytes with age.
- Tauopathy is associated with simpler initial morphology followed by the appearance of a cluster of complex cells at advanced stage.

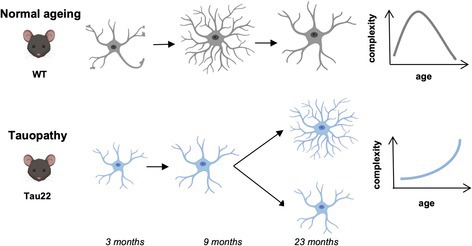

## 1 Introduction

Astrocytes are a major type of central nervous system (CNS) glial cells that regulate the function of neural circuits in the developing and adult brain. Along with microglia and oligodendrocytes, astrocytes also contribute to the onset, progression, and pathology in various neurodegenerative disorders (Khakh and Sofroniew 2015; Brandebura et al. 2023). Most of the functions performed by astrocytes rely on a close anatomical relationship with neurons and non‐neuronal cells in their local environment. Various labeling approaches have been developed to elucidate the complexity of their morphology (Baldwin et al. 2024). They have shown that astrocytes are defined by primary branches emanating from the soma, branchlets stemming from branches, and leaflets representing terminal processes. Their branches or leaflets are in close contact with pre‐ and post‐synaptic elements. In the hippocampal Cornu Ammonis 1 *stratum radiatum* (CA1sr), 80% of synapses are in contact with leaflets and 20% with branches (Gavrilov et al. 2018). The vast majority of astrocytes, if not all, have endfeet processes that interact with blood vessels (Kacem et al. 1998; Hosli et al. 2022), thereby regulating the blood–brain barrier (BBB), cerebral blood flow, nutrient uptake, and waste clearance (Diaz‐Castro et al. 2023). This unique proximity to neuronal circuits and blood supply provides them with an essential role in information processing and network wiring (Nagai et al. 2021; Zhou et al. 2021; Bonvento and Bolanos 2021), highlighting the importance of their integrity for a healthy brain function.

In recent years, single cell approaches have unveiled broad molecular, morphological, and physiological heterogeneity of astrocytes depending on brain regions and physiological contexts (Ben Haim and Rowitch 2017; Khakh and Deneen 2019). In the healthy mouse adult hippocampus, several morphological subtypes have been identified in the Dentate Gyrus (Karpf et al. 2022) as well as in the CA1 area (Viana et al. 2023). In addition to this pre‐existing diversity, a number of physiological and pathological conditions promote structural plasticity of astrocytes (Lawal et al. 2022; Oliet and Piet 2004). Aging is known to induce structural changes through mechanisms as yet unclear. A number of studies in mice have reported increasing astrocyte morphological complexity with age in some subregions of the hippocampus and *pars compacta* of the *substantia nigra*, accompanied by an increase in process length, cell volume, and number of branching points (Rodriguez et al. 2014; Bondi et al. 2021, 2023; Grosche et al. 2013; Rodriguez‐Callejas et al. 2023), whereas others claimed an opposite effect (Diniz et al. 2016; Popov et al. 2021). Many of these morphological studies were solely based on the immunostaining of the glial fibrillary acidic protein (GFAP), an intermediate filament, which only stains the major processes. In contrast, dye‐microinjection or fluorescent protein expression fills the entire cell and allows visualizing the fine processes of astrocytes, which may explain these discrepant observations.

Neurodegenerative conditions also affect astrocytes (Lee et al. 2022); however, little is known about the astrocytic response in primary tauopathies. This family of neurodegenerative diseases is characterized by the pathological aggregation of the tau protein not only in neurons but also in glial cells in the vast majority of cases. For example, tau‐positive astrocytic plaques are characteristic of corticobasal degeneration (CBD) whereas tau‐positive tufted astrocytes are a hallmark of progressive supranuclear palsy (PSP) (Kovacs 2020). While a growing body of evidence suggests that the accumulation of tau in astrocytes is not due to local upregulation of its expression (Ezerskiy et al. 2022; Forrest et al. 2023; Fiock et al. 2023) but is rather secondary to neuronal pathology, other studies show that changes in the local expression of microtubule‐associated protein tau (*Mapt*) may also contribute (Forrest et al. 2023). Astrocytes readily take up both filamentous and monomeric tau via a number of mechanisms, including binding to heparan sulfate proteoglycans, LRP1, integrin αV/β1 receptor, or via extracellular vesicles (Puliatti et al. 2023; Rauch et al. 2020; Perbet et al. 2023; Perea et al. 2019; Wang and Ye 2021). Since they also express phagocytic receptors such as MERTK and Multiple EGF‐like domain 10 (Megf10) (Chung et al. 2013; Tasdemir‐Yilmaz and Freeman 2014; Lee et al. 2021), it is likely that they ingest tau‐bearing synapses and cell debris for degradation (Taddei et al. 2023; Tzioras et al. 2023). Irrespective of the mechanism of penetration into the cell, tau seeds might then escape degradation and react with the endogenous pool of astrocytic tau to trigger further aggregation of tau (Mothes et al. 2023). By selectively expressing tau seeds and tau monomer in astrocytes and neurons, respectively, we recently confirmed that astrocytes can release tau seeds that eventually interact with neuronal tau and trigger the formation of both neuronal and astrocytic tau aggregates (Maté de Gérando Anastasie et al. 2021). The gradual exposure to pathological forms of tau triggers a number of molecular changes in astrocytes. We observed that astrocytes located in the *subiculum* that were exposed to hyperphosphorylated forms of tau are prone to degeneration (Maté de Gérando Anastasie et al. 2021). Altogether, these findings indicate that astrocytes develop responsive and/or degenerative molecular alterations in the presence of tau pathology. Since the spatial arrangement of astrocytes in relation to surrounding neurons is intimately linked to the accomplishment of their homeostatic functions, it is probable that tau pathology triggers morphological changes in astrocytes. Yet, this question has never been fully addressed.

Here, we explored the morphological plasticity of astrocytes in the context of a pure tauopathy, to better understand the astrocytic impact of pathological tau as pathology progresses. To that purpose, we implemented efficient tools—including a hierarchical clustering based on 10 morphological features—to define astrocytic complex morphology at different time points in the Thy‐tau22 (hereafter Tau22) transgenic mice. This mouse model expresses the human 4‐repeat tau mutated at sites G272V and P301S under a Thy1.2 promoter, driving human tau expression primarily into neurons. With age, these mice progressively develop tau‐related neuropathological changes, including tau hyperphosphorylation and neurofibrillary‐like tau inclusions at 9 months, as well as fibrillary tau inclusions in astrocytes detectable from the age of 12 months, concomitantly to synaptic loss (Maté de Gérando Anastasie et al. 2021; Schindowski et al. 2006). Here, our data confirm that hippocampal astrocytes in Tau22 mice progressively accumulate hyperphosphorylated tau. Interestingly, while at the very early stages, astrocyte morphology first exhibits a simplified profile of ramification, it develops into higher complexity profiles for a significant fraction of cells at very late stages of disease. This strongly contrasts with the bell‐shaped pattern of complexity observed in wild‐type (WT) animals. It suggests that the development of tauopathy associates with profound and complex changes in astrocytic morphological plasticity.

## 2 Materials and Methods

### 2.1 Animals

Seventeen male transgenic heterozygous Tau22 and WT littermate mice (2‐ [*n* = 6], 8‐ [*n* = 6], and 22‐ [*n* = 5] months old) were used in this study. All animal studies were conducted according to the French regulations (EU Directive 86/609 French Act Rural Code R 214‐87 to 131). The animal facility was approved by veterinarian inspectors (authorization n°B 92‐032‐02) and complies with the Standards for Humane Care and Use of Laboratory Animals of the Office of Laboratory Animal Welfare (OLAW: n#A5826‐01). All procedures received approval from the local ethical committee (Comité d'Ethique en Expérimentation Animale [CEA]) and the French Ministry of Research (APAFIS#22217‐2019100109316581 v1). Animals were grouped by five or fewer and housed in a temperature‐controlled room maintained on a 12 h light/dark cycle. Food and water were available ad libitum, and nesting material was added to the cage.

### 2.2 AAV Vector Construction and Production

For the labelling of hippocampal astrocytes, we designed a construct including tdTomato under the control of the astrocyte‐specific short gfaABC_1_D promoter, which was inserted into an AAV shuttle plasmid using Gateway recombination cloning technology (Invitrogen). For AAV production, this recombinant AAV genome was packaged into PHP.eB capsids by the MIRCen viral production platform as described (Berger et al. 2015). Vector concentration was assessed by quantitative PCR and expressed as viral genomes per milliliter (Vg/mL).

### 2.3 Injections of AAV Vectors

For systemic AAV administration, mice were maintained anesthetized by inhalation of a 2% isoflurane/O_2_ mixture. A 50 μL aliquot of the AAV vector preparation containing 2.5 × 10^10^ Vg was injected intravenously into the retro‐orbital sinus of mice using a 1‐mL syringe with a 30 G‐8 mm needle (BD microfine, Ref 324893 Becton Dickinson France) over 20–30 s.

### 2.4 RNA‐Sequencing Studies for the Quantification of *Mapt* Expression in Astrocytes

RNA‐sequencing studies were performed either on bulk hippocampal tissues (*n* = 4/group) or isolated astrocytes from the hippocampus (*n* = 3/group) from WT and Tau22 mice at 9 months of age (Paiva et al. 2024). RNA‐seq datasets generated in Paiva et al. are available at NCBI GEO under the accession numberGSE246777. The sequencing data was aligned on the human‐specific (129 bp) and murine‐specific (92 bp) *Mapt* gene using STAR v.2.5.3a, and the number of reads was counted using HTSeq‐count v.0.6.1p1. The number of reads covering the total *Mapt* gene (including both human and murine forms) was also counted in each sample. The number of reads was normalized and divided by the median of transcript length (in kb).

Murine *Mapt*‐specific sequence (92 bp from ENSMUSG00000018411):

AGCAGGCATCGGAGACACCCCGAACCAGGAGGACCAAGCCGCTGGGCATGTGACTCAAGCTCGTGTGGCCAGCAAAGACAGGACAGGAAATG.

Human *Mapt*‐specific sequence (129 bp from ENSG000000186868): GTACGGGTTGGGGGACAGGAAAGATCAGGGGGGCTACACCATGCACCAAGACCAAGAGGGTGACACGGACGCTGGCCTGAAAGAATCTCCCCTGCAGACCCCCACTGAGGACGGATCTGAGGAACCGGG.

### 2.5 Tissue Sectioning and Immunofluorescence

One month after injection, mice received a lethal dose of pentobarbital (ExagonVR, Axience, 400 mg/kg) by intraperitoneal injection. Animals then underwent intracardiac perfusion of 0.9% NaCl followed by 4% paraformaldehyde in 1X PBS. Brains were collected, post‐fixed overnight in 4% paraformaldehyde at +4°C. Then one hemisphere was cut into 40 μm sagittal sections using a freezing microtome (Leica Microsystems, Wetzlar, Germany) for AT8/SOX‐2 or GFAP/SOX‐2 immunofluorescence, while the other hemisphere was cut into 100 μm sagittal sections using a vibratome (Leica Microsystems, Wetzlar, Germany) for 3D reconstruction of astrocytes.

For AT8/SOX‐2, GFAP/SOX‐2, NeuN, or ThioS/SOX‐2 free‐floating immunofluorescence, 40 μm sections were incubated directly in 4.5% normal goat serum‐PBS 0.01 M‐ 0.2% Triton X‐100 (Sigma, Darmstadt, Germany) blocking solution before transfer into primary antibody solutions (AT8 MN#1020, 1/400 Thermo Fisher Waltham, MA, USA; SOX‐2 # PA1_094, 1/1000, Invitrogen, Waltham, MA, USA; GFAP‐AF488 644,704, Biolegend; NeuN MAB377, 1/1000 Merck Millipore, Burlington, MA, USA) for incubation overnight at +4°C. After rinsing three times in PBS 0.01 M—0.2% Triton X‐100, sections were transferred into Alexa‐coupled secondary antibodies diluted to 1/1000 in 3% normal goat serum‐PBS 0.01 M—0.2% Triton X‐100 for incubation 1 h at room temperature. They were then rinsed three times for 10 min with 0.01 M PBS—0.5% Triton X‐100, and incubated once for 10 min at room temperature in 1/5000 DAPI‐ 0.01 M PBS solution (62,248, Thermo Scientific, Waltham, MA, USA).

For tdTomato visualization and astrocyte 3D reconstruction, 100 μm sections were incubated first in 4.5% normal goat serum‐ 0.01 M PBS—0.5% Triton X‐100 (Sigma, Darmstadt, Germany) blocking solution for 72 h at +4°C, followed by a second blocking solution prepared with Mouse on Mouse Blocking Reagent (Vector MKB2213, Newark, CA, USA) diluted to 1/100 into 0.01 M PBS—0.5% Triton X‐100 solution for 2 h at room temperature. After rinsing three times with 0.01 M PBS—0.5% Triton X‐100, sections were incubated into AT100 (1/500) primary antibody solution for 48 h at +4°C. After rinsing 3 times for 30 min in 0.01 M PBS, sections were transferred into goat anti‐mouse AF488 coupled secondary antibody diluted to 1/500 into 0.01 M PBS—0.5% Triton X‐100 for incubation overnight at +4°C. They were then rinsed three times for 10 min with 0.01 M PBS—0.5% Triton X‐100, and incubated once for 10 min at room temperature in 1/5000 DAPI‐ 0.01 M PBS solution (62,248, Thermo Scientific, Waltham, MA, USA). Finally, sections were rinsed in 0.9% NaCl for 10 min and mounted on Superfrost slides (Menzel Gläser, polysine slides, Thermo Scientific, Waltham, MA, USA) and left to dry. They were then incubated in 0.06% KMnO_4_, rinsed for 10 min in 0.9% NaCl, and mounted in Vectashield HardSet mounting medium (H‐1400, Vector laboratories, Burlingame, CA, USA) covered by high precision microscope cover glasses (0101222, Marenfield Superior, Germany).

### 2.6 Image Acquisition and Image Analysis for Quantification of Immunofluorescence

A mosaic image of the whole CA1‐*subiculum* region on one 40 μm section was taken for each mouse using a Leica SP8 scanning confocal microscope, with a ×40 oil objective (NA = 1.4) at 581, 520, and 668 nm emission wavelengths for visualization of tdTomato/GFAP‐AF488/SOX‐2‐AF647, respectively. On a distinct series of sections, similar images were taken to visualize tdTomato/AT8‐AF 633/SOX‐2‐AF488 at 581, 647, and 520 nm. On average, 27 stacks were collected with a 1 μm step size. Using the Imaris (Bitplane, version 9.1 Belfast, UK) image analysis software, regions of interest were drawn manually in the CA1 pyramidal layer, CA1 *stratum oriens* + *stratum radiatum*, and *subiculum*.

To quantify global tau pathology within each subregion of interest, we applied a threshold on AT8 immunostaining and used the “surface” tool to calculate the percentage of AT8‐immunopositive volume.

To quantify the intensity of AT8 immunostaining within tdTomato‐positive cells, again we used Imaris to create a mask to detect tdTomato‐positive cells with their major processes. We then applied this mask to the channel displaying the AT8 immunostaining to extract the intensity sum of the AT8 signal and divided it by the tdTomato‐positive volume to obtain a normalized value of AT8 labeling for each mouse.

To quantify the percentage of AT8‐positive within the SOX‐2‐ positive astrocyte population, we used Imaris to create an AT8 “surface,” by thresholding the AT8 signal corresponding to what we considered to be a positive amount of labelling. This threshold was consistent for all mice and resulted in virtually no selected objects in WT mice, in which little or no AT8 labelling was expected. For each mouse, we then filtered the SOX‐2 surface with the “shortest distance to surface AT8” set to 0, to obtain the number of AT8‐positive cells amongst all SOX‐2‐positive cells.

To quantify the percentage of astrocytes displaying clasmatodendrosis within the SOX‐2 astrocyte population, we manually counted the number of astrocytes showing fragmented SOX‐2 immunostaining indicative of clasmatodendrosis on the whole hippocampal section, including the Dentate Gyrus.

### 2.7 Image Acquisition and Processing for 3D‐Reconstruction of Astrocytes

Single 2048 × 2048 pixel images were taken on a Leica SP8 scanning confocal microscope, using a 63x oil objective (NA = 1.4) at 581 nm emission wavelength. Z‐stacks were collected with a 0.3 μm step size throughout the entire section in order to include the whole territory of astrocytes. Only whole astrocytes were included in the analysis. Images were taken in the *stratum oriens* and *stratum radiatum* layers of the CA1 hippocampal dorso‐medial region and in the subiculum, in order to collect around 20 whole astrocytes per mouse per subregion. Images were then deconvoluted using AutoQuant X3 (10 iterations of deconvolution with high level of noise setting). 3D reconstructions for single astrocytes were completed with Imaris (Bitplane, version 9.1 Belfast, UK). Images were pre‐treated with a median filter (3*3*1) and the arborization of individual astrocytes was reconstructed using the filament tracer module and the “default autopath no loop” mode. Starting point diameter was set for each individual cell by manually measuring the soma diameter (calculated from the average of soma diameter on two orthogonal axes across the soma). Seed point diameter was set at 0.5 μm. The medians of the following morphological features were extracted for individual cell using the “statistics” tab: Total processes length, Process branch depths (Branch depth is defined as the number of branch points, or bifurcations, in the shortest path from the beginning point to a given point in the dendritic graph), Process branch Levels, Total number of branches, Total number of branching points, Median of Sholl intersections, Number of intersections at Sholl peak, number of Sholl intersection for every 1‐μm‐radius. The number of primary branches was estimated from the value of intersections at radius 1 μm. The Ramification index was calculated as the ratio of the number of intersections at Sholl peak profile over the number of primary branches.

### 2.8 Statistics

Statistical analysis was conducted using Statistica 13 software (Statsoft, Tulsa, OK, USA). Prior to analysis, the data were assessed for normality and homogeneity of variance. If it fulfilled the criteria for the general linear model, it was analyzed by one‐way analysis of variance (ANOVA) followed by Dunnett's or Bonferroni's post hoc tests for pairwise comparisons. The log10 transformation was used to normalize the data when necessary in order to perform statistical analysis. For the comparison of morphological features between WT and Tau22 mice, the data were analyzed with nested ANOVA to take into account individual mice as a source of variance.

### 2.9 Hierarchical Clustering

A clustering model was used to differentiate astrocyte subpopulations. The model was based on analysis of variance with a priori dimension reduction, followed by clustering analysis and finally linear discriminant analysis. Briefly, the data were first normalized for all variables using the *scale* function in the R base package (v 4.3.1), then principal component analysis (PCA) was applied as a preprocessing step before performing clustering using the prcomp function (stats package v. 4.3.1) (Josse 2012).

Two methods were used to determine the appropriate number of principal components (PCs): Kaiser's criterion and Horn's parallel analysis. PCs with eigenvalues greater than 1 in both analyses were considered to be components explaining sufficient variance in the data and retained for clustering analysis. Kaiser's criterion and Horn's parallel analyses both determined that the first 4, 3, and 3 PCs sufficiently explained the variance of 3‐, 9‐, and 23‐month‐old mice datasets, respectively (Figure S3). A comparison of k‐means, hierarchical, self‐organizing map (SOM) and partition around medoids (PAM) clustering algorithms on the retained PCs was performed using the clValid package (v 0.7) to identify the best clustering approach. The number of clusters was selected using the NbClust package (v. 3.0.1), which provides 30 indices to determine the optimal number of clusters in the data. Variables with a contribution > 0.7 in at least one cluster and a significant difference between clusters were considered for cluster comparisons. To validate the partition from the clustering analysis, a discriminant analysis of principal components (DAPC) was applied to the retained variables. This method uses an unsupervised learning classification algorithm based on a previous cluster analysis (Jombart et al. 2010). DAPC was performed using the *dapc* function from the adegenet package (v2.1.10).

Kruskal–Wallis test followed by Dunn's test with Benjamini‐Hochberg (FDR) correction was used to compare variables between clusters. All data analyses were performed in R version 4.3.1.

## 3 Results

Time‐course of neuronal and astrocytic pathology in the hippocampus of Thy‐Tau22 mice.

**FIGURE 1:**
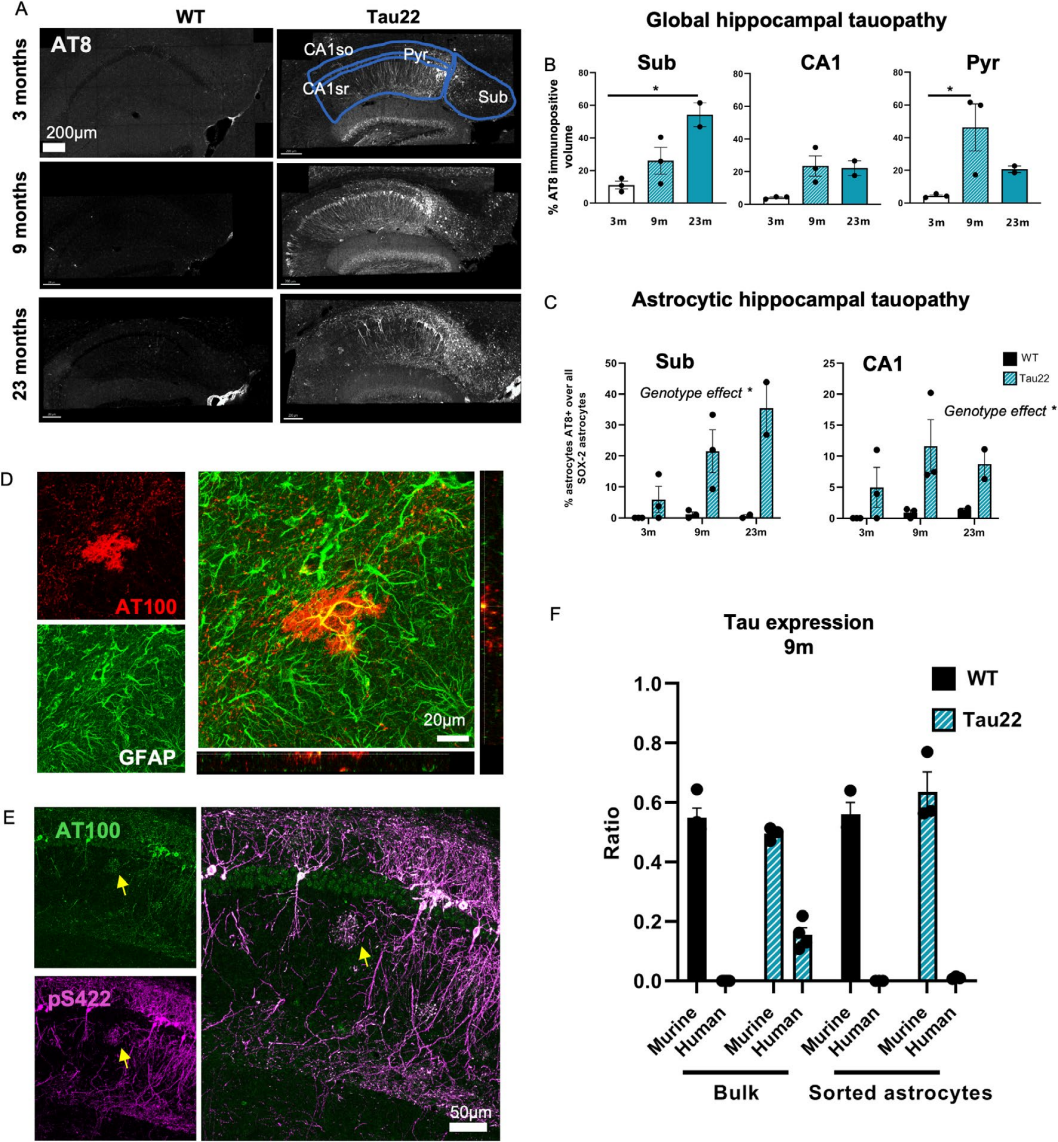
Time‐course of neuronal and astrocytic pathology in the hippocampus of Tau22 mice. (A) Representative images of AT8 immunofluorescent staining performed on hippocampal sections of 3‐, 9‐ and 23‐month‐old Tau22 mice and their WT littermates. Pathology was detectable early in the subiculum from 3 months of age, before the CA1 pyramidal neurons also became affected at 9 months of age. At 23 months old, robust tau pathology was observed in the subiculum while that in CA1 seemed to decrease, possibly due to neuronal loss. (B) The percentage of AT8 immunostained volume within a 40 μm‐thick section of the hippocampus was quantified, in the CA1 so/sr subregions, the CA1 pyramidal layer and the subiculum for the three age groups in Tau22 mice. A significant evolution of pathology over time was observed in the CA1 pyramidal layer and the subiculum. (C) Quantification of the percentage of AT8/SOX‐2‐immunopositive astrocytes within the SOX‐2 positive population in a 40 μm‐thick section of the hippocampus in the CA1 region and the subiculum for the 3 age groups in Tau22 mice. (D) Example of GFAP/AT100 immunofluorescent staining in the CA1 region in an 18‐month‐old Tau22 mouse. (E) Representative image of p‐Ser422/AT100 immunofluorescent staining in the CA1 region in a 23‐month‐old Tau22 mouse. Many scattered CA1 pyramidal neurons were immunopositive for both markers of tau pathology. Tau‐positive astrocytes were also detected in close proximity to neuronal dendrites in the stratum radiatum layer (yellow arrow). They displayed strong tau labelling in their major processes as well as in their terminal points, with fainter labelling of their soma. (F) Murine and human expression of tau (Mapt) determined by RNA‐seq analysis in samples from hippocampal bulk tissue or sorted astrocytes of WT and Tau22 mice at the age of 9 months. Expression is shown as the ratio of tau of interest (murine or human) over total tau (murine and human). Data were analyzed with a one‐way ANOVA (B), two‐way ANOVA (C, for CA1) or non‐parametric Mann–Whitney test (C for Sub) with age and genotype as factors. Significant comparisons (*p* < 0.05) are indicated by an asterisk in B and C. All data are presented as mean ± SEM, with *n* = 2–3 mice per genotype. CA1, Cornu Ammonis 1; Sub, subiculum; Pyr, pyramidal layer of CA1.

We previously reported that while tau pathology initiates in neurons in Tau22 mice, it is followed by an accumulation of tau aggregates in astrocytes in various subregions of the hippocampus (Maté de Gérando Anastasie et al. 2021), suggesting that astrocytes are progressively exposed to pathological forms of tau arising from neuronal cells. To further assess the time course of tau pathology in this model, we first evaluated the percentage of positively stained tissue volume using the AT8 antibody that detects tau hyperphosphorylation in 3‐, 9‐, and 23‐month‐old male transgenic Tau22 mice. AT8 immunolabeling increased sharply between 3 and 9 months in the CA1 pyramidal layer containing pyramidal neurons (Pyr), the surrounding layers containing their dendrites (including *stratum oriens* [CA1so] and *stratum radiatum* [CA1sr], hereafter named CA1so‐sr) and in the adjacent *subiculum* (Sub), receiving CA1 neuron inputs (Figure 1A,B). By 23 months, pathology tended to decrease in the pyramidal layer, presumably because of neuronal death, while increasing further in the subiculum. Our previous observations of tau accumulation in Tau22 astrocytes prompted us to quantify the percentage of affected astrocytes as pathology progresses. To that end, we carried out a double immunolabeling for AT8 and SOX‐2, a nuclear transcription factor expressed in over 97% of mature post‐mitotic astrocytes in the adult hippocampus (Jinno 2011). We then measured the percentage of AT8‐positive/SOX‐2‐positive astrocytes over the whole SOX‐2‐positive population and found that it was higher in Tau22 mice compared to WT littermates, reaching up to 40% of subicular astrocytes in 23‐month‐old Tau22 mice (Figure 1C and Figure S1A).

Double immunostaining with GFAP and AT100 (Figure 1D) or pSer422 (Figure 1E) antibodies detecting distinct pathological epitopes also labeled CA1 neurons and astrocytes. In contrast, ThioS staining of mature fibrillary aggregates was positive in Pyr neurons but could not be detected in astrocytes at any age (Figure S2A). We next wondered whether tau accumulation in astrocytes could be attributed to increased *Mapt* expression within these cells. To address this question, we performed a new analysis on RNA‐seq datasets generated from hippocampal sorted astrocytes and bulk tissue in 9‐month‐old Tau22 mice (Paiva et al. 2024). Through the use of species‐specific sequences, we were able to differentiate murine and human mutant tau expression. As expected, murine *Mapt* expression was seen in all WT and Tau22 samples at the same level (Figure 1F). Expression of the human *Mapt* transgene was detected only in Tau22 mouse samples when samples were prepared from bulk hippocampal tissue, whereas no expression of this gene was detected in samples from isolated hippocampal astrocytes, showing that the Thy1 promoter‐dependent transgene is not expressed in astrocytes of Tau22 mice.

From 9 months of age, we noted the progressive appearance of beaded SOX‐2 immunostaining, reminiscent of clasmatodendrosis and suggesting astrocyte degeneration (Figure S1B). We determined the % of such “fragmented” astrocytes over the whole SOX‐2 population in Tau22 mice and their littermates. This fraction increased significantly from around 0.1% at 9 months to 3%–4% at 24 months; however, there was no effect of genotype, suggesting that this phenomenon is likely linked to aging rather than to tau pathology (Figure S1C).

## 4 Labelling of Hippocampal Astrocytes for 3D‐ Reconstruction and Analysis of Morphology

**FIGURE 2:**
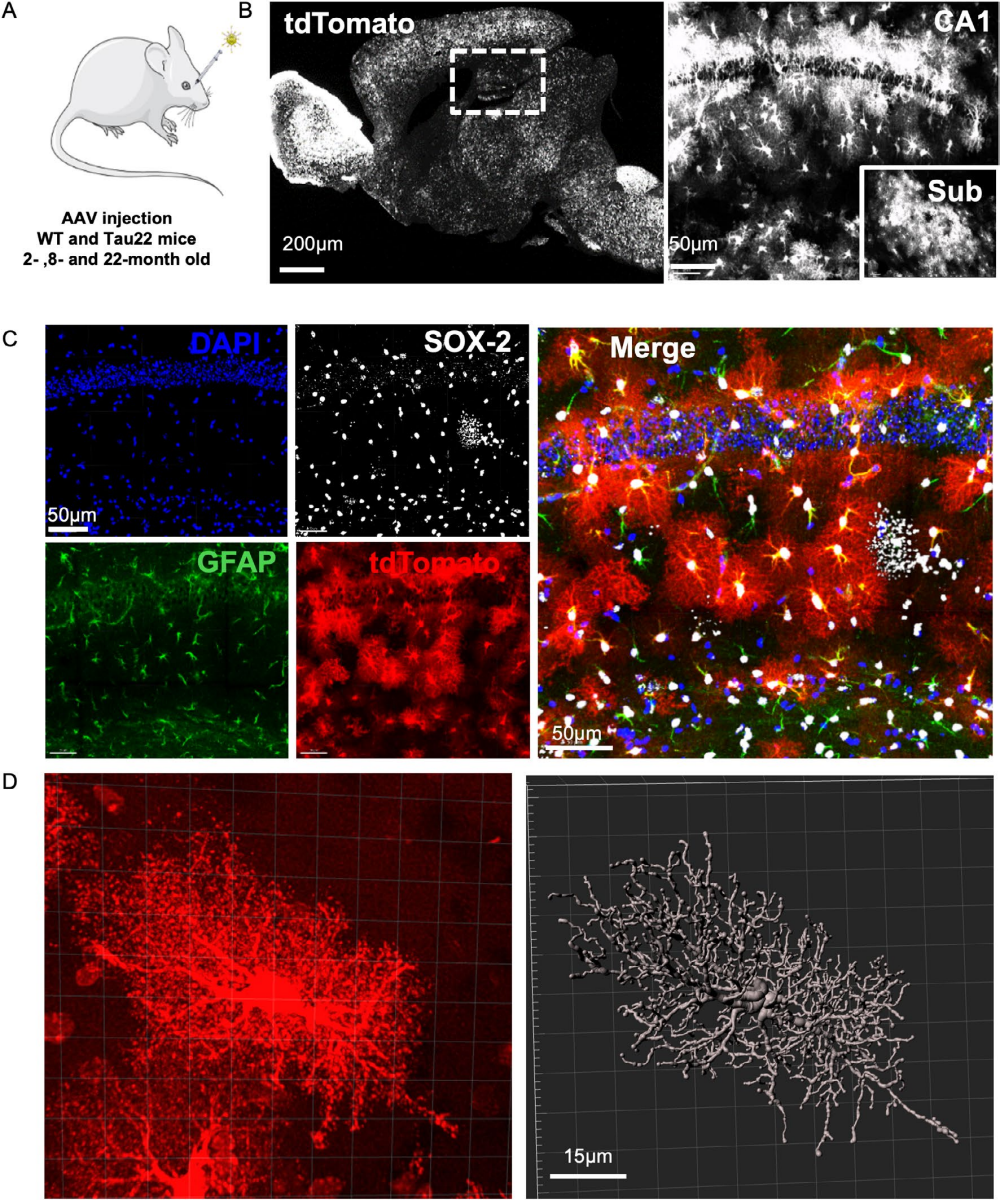
Labelling of hippocampal astrocytes for 3D reconstruction and analysis of morphology. (A) Experimental design of the morphological analysis of hippocampal astrocytes. 2‐, 8‐, and 22‐month‐old Tau22 male mice and their WT littermates were injected with an AAV encoding fluorescent tdTomato reporter gene under an astrocytic promoter. (B) Sparse astrocytic labelling was observed a month later in the whole brain, including the CA1 region of the hippocampus and the subiculum. (C) Fluorescent immunolabeling for SOX‐2, GFAP, tdTomato, and DAPI showing various astrocyte populations labeled with some or all of these astrocyte‐specific markers in the CA1 region. (D) A representative image of a tdTomato‐positive astrocyte and its corresponding Imaris‐3D reconstruction used for the extraction of morphological measurements. CA1, Cornu Ammonis 1; Pyr, CA1 pyramidal layer; Sub, subiculum.

Since astrocytes become increasingly exposed to pathological forms of tau in aging Tau22 mice, we investigated how their morphology changes over time. To perform an extensive analysis of astrocyte morphology, we injected Tau22 mice and their littermate controls at 2, 8, and 22 months intravenously (retro‐orbitally) with an AAV‐PHP.eB capsid enabling BBB crossing and driving the expression of tdTomato fluorescent protein under a short GFAP promoter (Figure 2A). One month later, 100 μm‐thick hippocampal sections were examined using confocal microscopy. This procedure achieved sparse labeling of astrocytes in the hippocampus to allow the 3D‐ reconstruction of the morphology of entire individual astrocytes (Figure 2B–D). As we performed a triple fluorescent immunolabeling for SOX‐2, GFAP, and tdTomato, we found that the entire population of astrocytes was SOX‐2‐immunopositive, followed by a large SOX‐2^+^/GFAP^+^ fraction and a smaller fraction of SOX‐2^+^/GFAP^−^ cells (Figure 2C). Importantly, all tdTomato^+^ cells were also SOX‐2^+^, demonstrating that reconstructed tdTomato cells were all astrocytes. We also took advantage of the tdTomato labeling to measure the intensity of AT8 immunostaining in the astrocytic cell body and major processes (Figure 2B,C). We observed that the AT8 signal was stronger in Sub and CA1 of Tau22 mice compared to WT controls (Figure 1C).

## 5 Astrocyte Morphology Acquires Complexity During Adulthood Before Simplifying at Old Age in WT Mice

**FIGURE 3:**
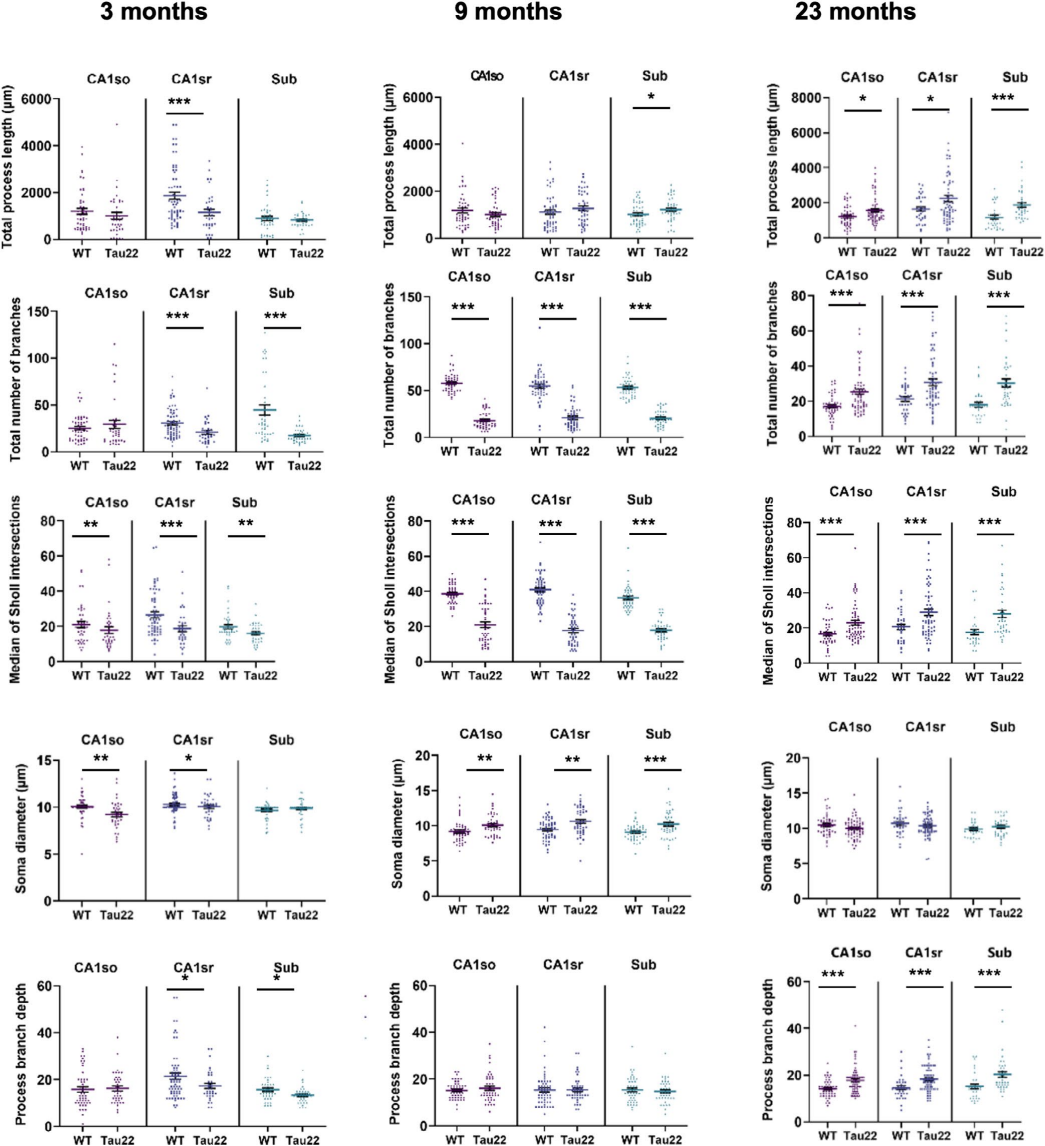
Multiple features of morphological complexity differentiate astrocytes exposed to tau pathology from WT astrocytes. A selection of morphological features from 3D‐reconstructed astrocytes is shown. They were compared between Tau22 mice and WT littermates at different ages. Data plotted are individual astrocyte values (dots) from 2 to 3 mice/group and mean ± SEM for each group. Data were analyzed with nested ANOVA to take into account individual mice as a source of variance. *p < 0.05, **p < 0.01, ***p < 0.001. Data plotted are individual astrocyte values (dots) from 2 to 3 mice/group and mean ± SEM for each group. Data were analyzed with one‐way ANOVA. *p < 0.05, **p < 0.01, ***p < 0.001. CA1, Cornu Ammonis; CA1so, CA1 stratum oriens; CA1sr, CA1 stratum radiatum; Sub, subiculum.

We then conducted a morphometric analysis on an average of 20 tdTomato‐positive astrocytes/mouse/subregion/age. In total, we compiled 817 reconstructions of astrocytes over three subregions, three ages, and two genotypes. Ten features covering a broad range of cell morphology were extracted for each astrocyte (Figure 3 and a comprehensive dataset in Tables S1 and S2). We did not attempt to reconstruct the morphology of fragmented astrocytes. Overall, there was minimal regional variability between CA1so, CA1sr, and *subiculum* for most parameters, especially in young animals. We first examined the effect of aging in WT mice (Figure S3). The soma diameter exhibited minimal variation over time, although there were significant changes. It initially decreased towards adulthood and then increased in old age. Conversely, the number of branches and the median of Sholl intersections displayed an opposite pattern of changes. The total process length evolved differently according to hippocampal subregions. It was stable in CA1so, while it increased continuously with age in the subiculum, and displayed an inverted bell‐shaped curve in CA1sr. The process branch depth remained stable over time.

## 6 Multiple Features of Morphological Complexity Differentiate Astrocytes Exposed to Tau Pathology From Wild‐Type Astrocytes

**FIGURE 4:**
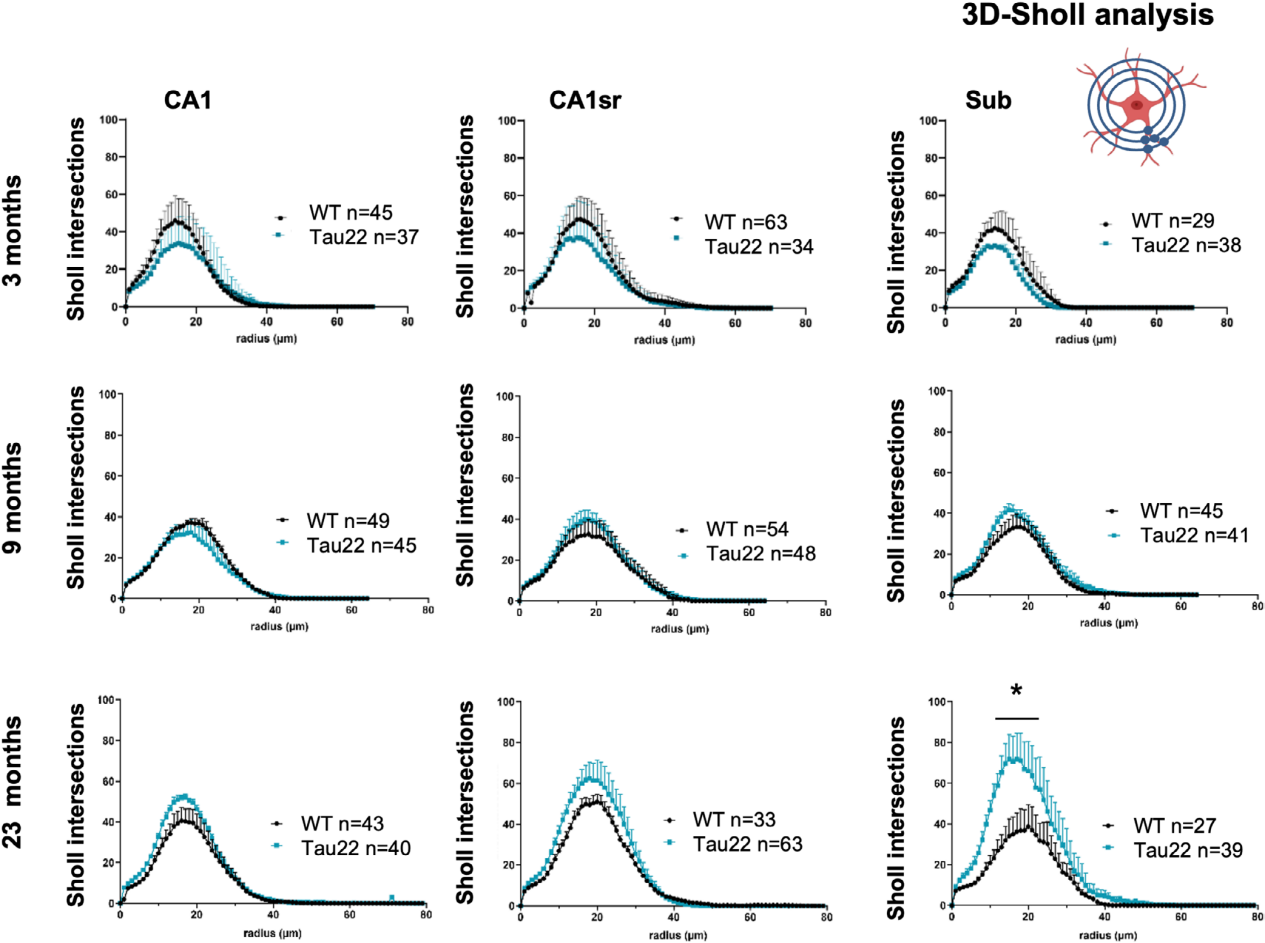
Astrocyte morphology changes with the progression of tau pathology. 3D‐Sholl analysis of tdTomato‐labeled astrocytes in the three subregions and for the three ages (*n* = 27 to 63 cells from 2 to 3 mice/genotype/age, as indicated). Tau22 subicular astrocytes in 23‐month‐old mice had significantly more ramifications than WT mice from radius 14–19 (two‐way ANOVA followed by Bonferroni's test *p* < 0.01). CA1, Cornu Ammonis; CA1so, CA1 stratum Oriens; CA1sr, CA1 stratum radiatum; Sub, subiculum. Plotted data are mean ± SEM for each group.

We also compared astrocytic morphology between WT and Tau22 mice and found that tau genotype altered astrocyte morphology differently at each stage of the disease (Figure 3 and Table S2). In 3‐month‐old mice, astrocytes from Tau22 mice tended to have a simpler morphology compared to their WT littermates, with fewer Sholl intersections, fewer branches, fewer branch levels, fewer branching points, and shorter branches. Interestingly, aging enhanced some of these differences at 9 months, particularly for the total number of branches and Sholl intersections, which increased in CA1sr and Sub in WT mice but not in Tau22 mice. The soma diameter enlarged slightly but significantly in Tau22 mice, evocative of reactive hypertrophy. At that stage, changes occurring in the subiculum started to show a different trend from the other subregions, with more primary branches and longer processes in Tau22 mice compared to their WT counterparts. Interestingly, at the final stage of the disease, other subregions eventually caught up with the subiculum, with an increased number of Sholl intersections, branch levels, number, and length, all indicating a significant complexification of the morphology of astrocytes in Tau22 mice compared to WT littermates. The comparison of average Sholl profiles between both genotypes further supported this phenomenon of complexification in Tau22 mice as the disease progresses, with a particularly strong response in the subiculum (Figure 4). In this subregion, the number of intersections at the peak of the curve occurred at the same radius but was almost double in 23‐month‐old Tau22 compared to WT mice.

## 7 Tau Pathology Remodels the Distribution of Morphologically Distinct Subpopulations of Astrocytes

**FIGURE 5:**
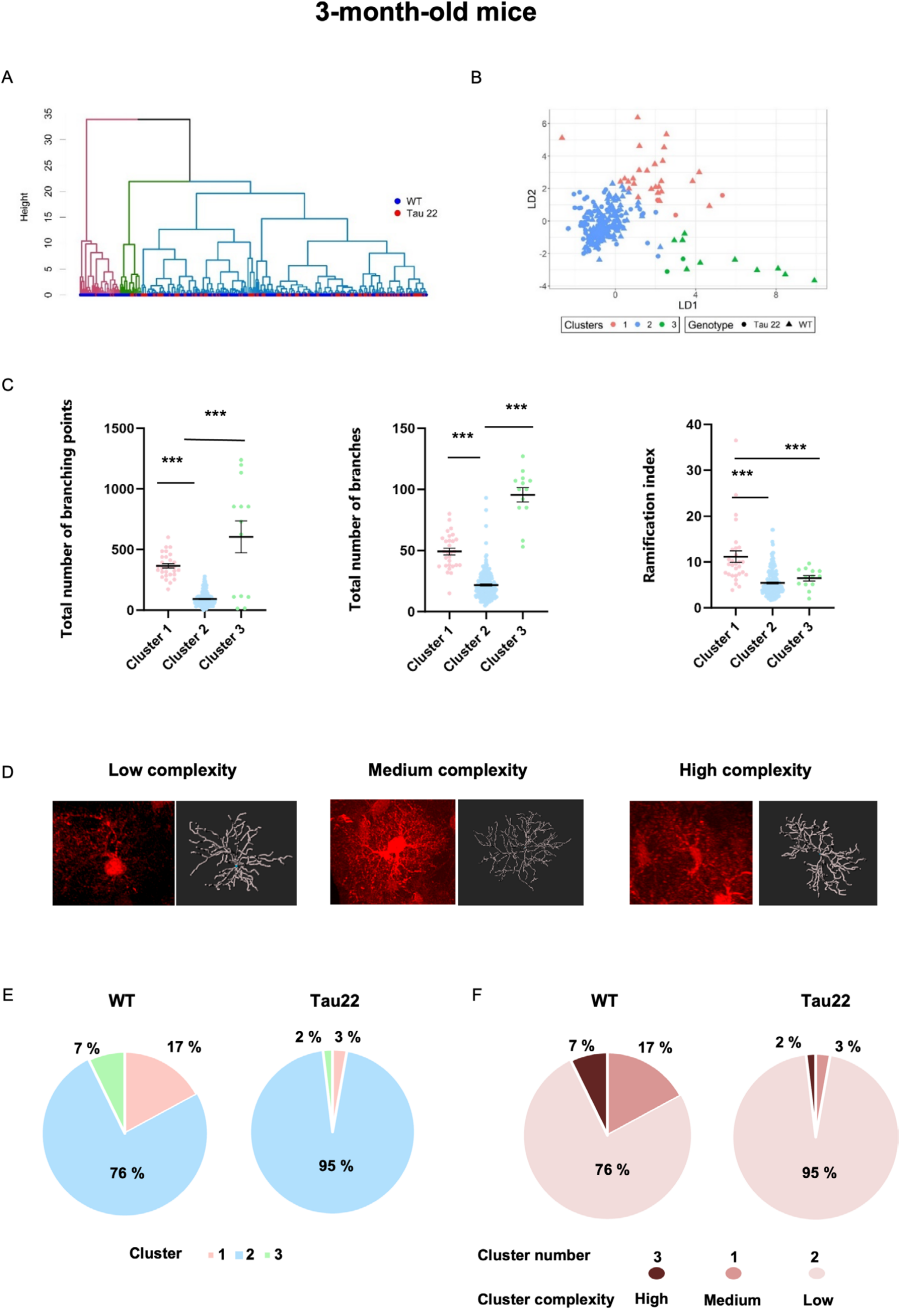
Classification of astrocytes according to their morphological features in 3‐month‐old mice. (A) Dendrogram of hierarchical clustering indicating clusters by color. (B) Plot of discriminant analysis. The dataset is clustered into three clusters. (C) Selection of morphological features for astrocytes in clusters 1–3 for 3‐month‐old mice. Data plotted are individual astrocyte values (dots) from 3 mice/group from all subregions together, and mean ± SEM for each group in black. Data were analyzed with Kruskal‐Wallis. ***p < 0.001. (D) Representative images of tdTomato‐labeled astrocytes and their 3D skeleton for each cluster of graded morphological complexity. (E) Proportions of clusters within the two genotypes WT and Tau22 represented as pie charts, and (F) color‐coded according to their score of morphological complexity in brown.

**FIGURE 6:**
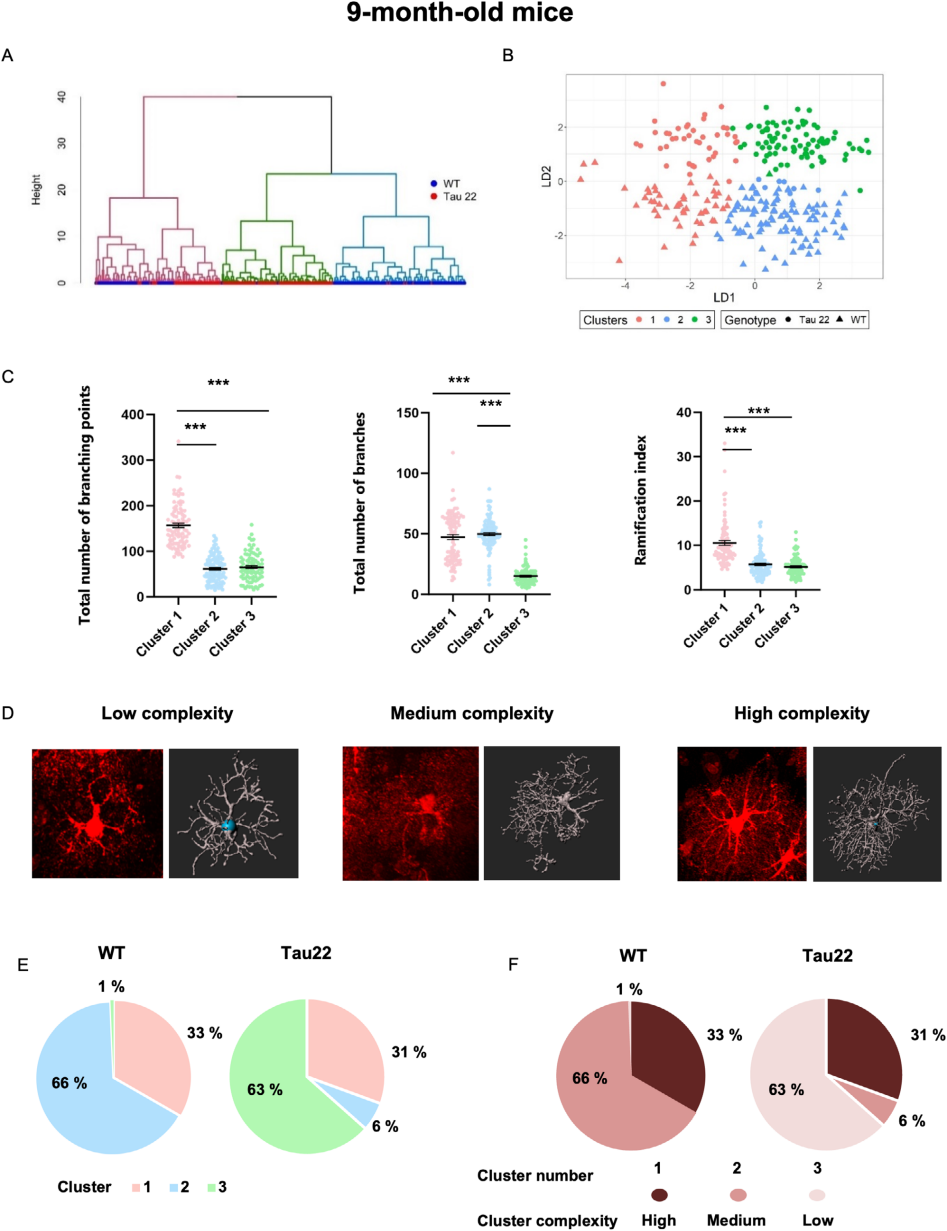
Classification of astrocytes according to their morphological features in 9‐month‐old mice. (A) Dendrogram of hierarchical clustering indicating clusters by color. (B) Plot of discriminant analysis. The dataset is clusterized into three clusters. (C) Selection of morphological features for astrocytes in clusters 1–3 for 9‐month‐old mice. Data plotted are individual astrocyte values (dots) from 3 mice/group from all subregions together, and mean ± SEM for each group in black. Data were analyzed with Kruskal‐Wallis. ***p < 0.001. (D) Representative images of tdTomato‐labeled astrocytes and their 3D skeleton for each cluster of graded morphological complexity. (E) Proportions of clusters within the two genotypes WT and Tau22 represented as pie charts and (F) color‐coded according to their score of morphological complexity in brown.

**FIGURE 7:**
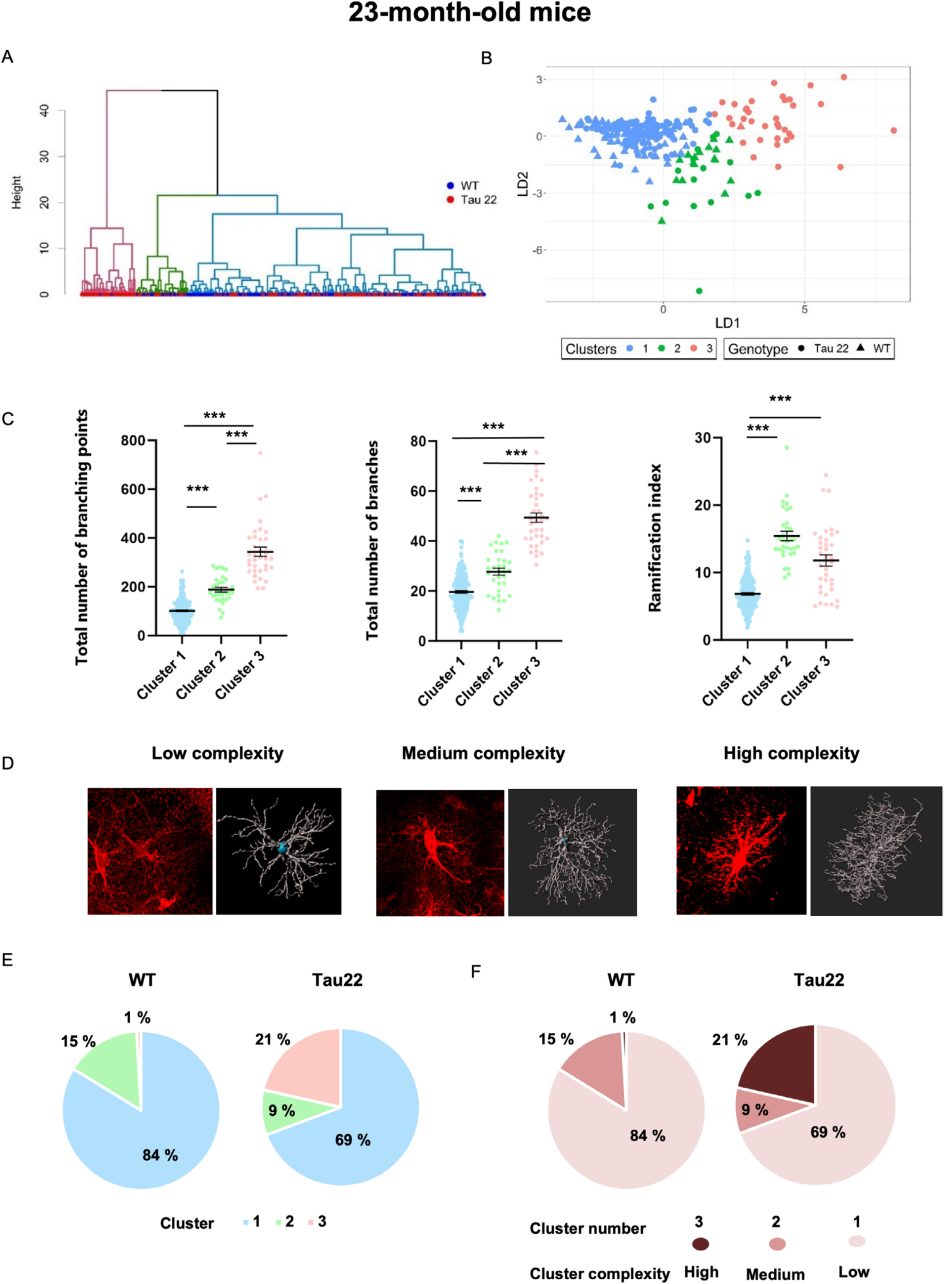
Classification of astrocytes according to their morphological features in 23‐month‐old mice. (A) Dendrogram of hierarchical clustering indicating clusters by color. (B) Plot of discriminant analysis. The dataset is clustered into three clusters. (C) Selection of morphological features for astrocytes in clusters 1–3 for 23‐month‐old mice. Data plotted are individual astrocyte values (dots) from 3 mice/group from all subregions together, and mean ± SEM for each group in black. Data were analyzed with Kruskal‐Wallis. ***p < 0.001. (D) Representative images of tdTomato‐labeled astrocytes and their 3D skeleton for each cluster of graded morphological complexity. (E) Proportions of clusters within the two genotypes WT and Tau22 represented as pie charts, and (F) color‐coded according to their score of morphological complexity in brown.

Recent transcriptomic studies have revealed a vast heterogeneity in the astrocyte population (Ben Haim and Rowitch 2017; Khakh and Deneen 2019; Endo et al. 2022), suggesting that within the hippocampus, morphologically and phenotypically distinct subpopulations may emerge in a pathological context. To address this question in the context of a pure tauopathy, we subjected our morphological dataset of each age group to a clustering model based on multivariate methods. For each of the three age groups, three clusters were identified (Figures 5A,B, 6A,B, 7A,B and Figure S4). Morphological parameters that exhibited significant differences between clusters were retained for cluster validation (hence “soma diameter” was excluded for the 3‐ and 23‐month‐old analysis, and “total number of primary branches” was excluded for 3‐ and 9‐month‐old analysis). We then improved the initial partition obtained from hierarchical clustering by applying a discriminant analysis of principal components on the values of the retained variables. It is important to note that because we performed 3 distinct hierarchical clustering, the morphotype of each cluster differs at each age (Figure 5C,D, 6C,D, and 7C,D), for example, the morphology of cells in cluster 1 in 3‐month‐old mice is different from that of cluster 1 in 9‐month‐old mice, despite the use of the same cluster nomenclature.

To determine how the distribution of astrocyte morphotypes changed with genotype, we calculated the percentage of cells belonging to each cluster and subsequently compared cluster distribution between Tau22 mice and their WT littermates at each age (Figures 5E, 6E, 7E). In parallel, we assigned a complexity score to each cluster (from “low” to “high” in graded brown charts in Figure 5F‐6F‐7F), with “high complexity” reflected by the combination of elevated numbers of branching points, total number of branches, and ramification index. Even from an early stage, cluster distribution was different between Tau22 and WT mice (Pearson Chi2 *p* < 0.0005). At 3 months of age, a large fraction of cells belonged to cluster 2 (95.3% and 75.7% respectively) exhibiting low complexity, followed by cluster 1 (2.8% and 17.1% respectively) typified by a relatively complex morphology and a minority to cluster 3 (1.9% and 7.2% respectively) (Figure 5E, Suppl. Figure 5). Hence, the proportion of fairly complex cells belonging to clusters 1 and 3 was reduced at an early stage of tauopathy in favor of cluster 2 cells of low complexity. At 9 months old (Figure 6E, Supplementary Figure 5), Tau22 and WT mice had the same % of cluster 1 cells (30.6% and 33.3% respectively) but displayed a striking difference regarding cluster 2 (6% and 66% respectively) and cluster 3 (63.4% and 0.7% respectively). Tau22 mice displayed mainly cluster 3 astrocytes with fewer Sholl intersections and a number of branches whereas WT mice exhibited mostly cluster 2 astrocytes with more ramifications. The distribution of astrocytes on a PC plane in Figure 6B clearly revealed the influence of Tau genotype on the 3 clusters' subpopulations (Pearson Chi2 *p* < 0.0001). Overall, these data showed that until 9 months of age, tau pathology tended to simplify the morphology of astrocytes. At 23 months old, 83.8% of WT astrocytes belonged to cluster 1 compared to 69.3% for Tau22 mice. This predominant cluster corresponded to the most simplified morphology (fewer Sholl intersections, fewer and shorter branches, fewer branch levels, Figure 7 and Supplementary Figure 5). Interestingly, Tau22 mice had 21.5% of their astrocytes belonging to cluster 3 (corresponding to the most complex cells) compared to only 0.9% in WT mice. In contrast, the proportion of cluster 2 cells of medium complexity was less different between genotypes (9.2% in Tau22 versus 15.3% in WT mice). Hence, in this final stage of the disease, a surprising process of morphological complexification seemed to occur in Tau22 mice, affecting a non‐negligible fraction of CA1 hippocampal astrocytes (Pearson *χ* ^2^ *p* < 0.0001).

## 8 Discussion

Astrocytes are capable of morphological plasticity to accomplish their physiological functions (Lawal et al. 2022; Oliet et al. 2004) but also to adapt to acute and chronic pathological conditions. Little is known about their morphological alterations at different stages of tauopathy, a neurodegenerative condition in which astrocytes play a decisive role. Here we show that in the hippocampus of Tau22 transgenic mice, as they become increasingly exposed to pathological tau, astrocytes accumulate hyperphosphorylated tau. Using cell‐specific targeted expression of a fluorescent reporter gene, we were able to sparsely label hippocampal astrocytes to perform their 3D‐reconstruction at three stages of pathology and extract a range of morphological features. Using hierarchical clustering, we revealed that tau pathology first induced a simplification process of their arborization, followed by the unexpected emergence of a significant cluster of complex cells at the final stage of disease.

In Tau22 mice, tau pathology begins in neurons, and we confirmed that this can be attributed to the highly selective neuronal specificity of the Thy1.2 promoter. In agreement with previous studies, we have shown that pathological hyperphosphorylated tau could be detected with the AT8 antibody from 3 months of age in the pyramidal CA1 neurons of the hippocampus and in the subiculum. It increased significantly, affecting more and more neurons at 9 months of age, to finally decline at the final stage of disease at 23 months old. This fading is consistent with the neuronal death reported by others in Thy‐Tau22 mice (Schindowski et al. 2006; Viney et al. 2022). As pathology progressed, we observed more AT8‐immunopositive astrocytes in close proximity to affected neurons in the CA1sr and CA1so layers containing their dendrites, as well as in their closest efferent output region, the subiculum (Berns et al. 2018). In order to quantify the proportion of affected astrocytes, we counted the number of AT8‐positive astrocytes within the SOX‐2^+^ astrocytic population. We found that a significant fraction of CA1 astrocytes displayed detectable nuclear and cytoplasmic tau immunostaining at the peak of neuronal pathology. In some cases, the whole arborization down to their nucleus was positively stained for AT8‐tau. In the normal brain, physiological levels of tau are found in the nucleus, where it protects DNA from damage and keeps heterochromatin condensed (Frost 2023). These functions may be lost in the presence of pathological species of tau and lead to aberrant gene expression and to lethal cell‐cycle re‐entry (Frost et al. 2014). Indeed, the number of senescent astrocytes increases through aging and AD (Bhat et al. 2012) and in Tau P301S transgenic mice (Bussian et al. 2018). The pharmacological removal of senescent astrocytes and microglia with senolytics almost completely prevents hyperphosphorylated tau and NFT deposition while neurodegeneration continues unabated (Bussian et al. 2018), strongly suggesting that some astrocytes acquire a phenotype deleterious for neurons. Recent transcriptomic analysis in PSP patients (Sharma et al. 2021; Briel et al. 2022), iPSC‐derived astrocytic cultures (Ezerskiy et al. 2022) and tau transgenic mice (Dejanovic et al. 2018; Litvinchuk et al. 2018; Jiwaji et al. 2022) have revealed astrocyte profiles associated with both deleterious and adaptive‐protective signals, compatible with the co‐existence of astrocytic sub‐types in tauopathies.

The origin of this astrocytic tau is unclear since the *Mapt* gene, which encodes tau, is normally poorly expressed in astrocytes (Zhang et al. 2014, 2016). Measurement of the tau protein by mass spectrometry in human tau (hTau) transgenic mice expressing all human tau isoforms revealed that astrocytes express 100‐fold less tau than neurons (Ezerskiy et al. 2022). Human single‐cell sequencing and RNA in situ hybridization data show divergent up‐ or down‐regulation of tau expression in astrocytes in PSP patients (Forrest et al. 2023; Fiock et al. 2023; Jackson et al. 2024). Here, we re‐examined RNA‐seq data sets of tissue taken from 9‐month‐old Tau22 mice (Paiva et al. 2024). We found no expression of human tau in sorted astrocytes, suggesting that increased accumulation of Tau in astrocytes is not linked to increased expression, at least in this transgenic model. One hypothesis put forward to explain the presence of astrocytic tau aggregates is that tau seeds are released from neurons into the extracellular space before being taken up by astrocytes. Several studies have shown that astrocytes take up various forms of pathological tau via a number of mechanisms, possibly involving phagocytosis (Puliatti et al. 2023; Rauch et al. 2020; Perbet et al. 2023; Perea et al. 2019; Wang and Ye 2021; Chung et al. 2013; Tasdemir‐Yilmaz and Freeman 2014; Lee et al. 2021). A study from our laboratory showed that following AAV‐induced neuronal expression of a pro‐aggregant form of tau, fewer tau aggregates are found in neurons and astrocytes of tau KO mice compared to WT littermates (Maté de Gérando Anastasie et al. 2021), supporting the idea that the small amount of astrocytic monomeric tau plays a role in tau seeding and the formation of astrocytic tau lesions.

We have performed an in‐depth morphological analysis of hippocampal astrocytes at different stages of severity of tauopathy, using tdTomato fluorescent labeling in 100 μm‐thick sections. This approach was, therefore, free from the bias associated with potentially variable expression of endogenous astrocytic markers such as GFAP, GS, or S100β. The thickness of the sections allowed us to visualize the entirety of the astrocytic arbor, from the soma down to the tip of processes (but excluding the tiny leaflets not accessible with conventional confocal microscopy) and to extract morphological features in three dimensions. The comparison of a panel of morphological variables between Tau22 mice and their WT littermates and the identification of morphological subtypes by hierarchical clustering analysis revealed a distinct bi‐phasic trajectory of changes for both groups. At all ages, we found three clusters of astrocytic morphotypes of graded complexity.

Physiological aging itself was accompanied by astrocytic morphological changes. In WT mice, we found that astrocytes developed an increasing number of branches from young (3 months old) to middle‐aged (9 months old) WT mice, but a sharp drop was then observed in the period between the ages of 9 and 23 months. The median of Sholl intersections declined continuously with age. Cluster analysis showed that the distribution of clusters of varying complexity also changed with time. A predominant subpopulation of medium complexity developed into more complex cells by 9 months. Yet at 23 months old, cells of intermediate complexity were replaced by a large fraction of simple cells. Altogether, our data show that astrocyte morphology in the hippocampus is dynamic throughout aging. Its overall complexity peaks at middle age and then declines at an advanced age. When compared with studies performed over the same age range, this trajectory is consistent with other data obtained in the Dentate Gyrus and the CA1 regions of mice (Bondi et al. 2021; Diniz et al. 2016; Popov et al. 2021) and in the frontal lobe and entorhinal cortex of nonhuman primates (Rodriguez‐Callejas et al. 2023; Robillard et al. 2016).

Little is known about the specific effect of tau pathology on astrocyte morphology. Here, we report a dual effect of tauopathy. Early on, before the appearance of major tau aggregation, the number of branch levels, Sholl intersections, branching points, and branches were all diminished in most subregions of Tau22 mice, suggesting a simpler morphology compared to WT littermates. These differences were exacerbated at 9 months as cell complexity continued to increase in WT mice but decreased in Tau22 mice. This phenomenon was particularly visible by clustering analysis, where the majority of cells belonged to the simplest morphotype in Tau22 mice, as opposed to the medium‐complexity cluster in WT mice. Interestingly, at 9 months old, both genotypes also had a third of their cells with the most complex morphotype. Unexpectedly, this particular cluster was conserved in 23‐month‐old Tau22 mice but had disappeared in WT mice. Thus, at that later stage, two main morphotypes coexisted in Tau22 mice: a small cluster characterized by long processes, with many branching points and levels, and a predominant cluster of much simpler morphology. Intriguingly, Togo and Dickson (Togo and Dickson 2002) also reported coexisting subtypes of reactive astrocytes in the brain of PSP patients. They found that GFAP was increased in astrocytes close to neurofibrillary tangles but diminished in tufted astrocytes with tau pathology. Similarly, astrocytes in age‐related astrogliopathy (ARTAG) also display atrophic processes with reduced glial GFAP and increased superoxide dismutase 2 immunoreactivity (Ferrer et al. 2018). Our observation that a fraction of hippocampal astrocytes exhibit AT8‐positive staining is consistent with the idea that tau‐positive and tau‐negative astrocytes coexist. It is unclear if the morphotypes we describe here are related to varying degrees of tau accumulation in astrocytes. This will be addressed in further studies combining tau‐immunolabeling and 3D reconstruction. Previous studies of morphological changes in other neurodegenerative mouse models have not fully addressed this heterogeneity. In 3xTG‐AD (carrying a mutation in three genes: APP K670_M67, PSEN1 M146V, and MAPT P301L), PDAPP‐J20 (PDGFB‐APPSwInd with a mutation in the APP gene) and APP/PS1dE9 (expressing a chimeric mouse/human amyloid precursor protein [Mo/HuAPP695swe] and a mutant human presenilin 1 [PS1‐dE9]) the astroglial volume, surface area, and complexity were found to be reduced overall (Endo et al. 2022; Olabarria et al. 2010; Yeh et al. 2011; Kulijewicz‐Nawrot et al. 2012; Beauquis et al. 2013; Rodriguez‐Arellano et al. 2016). Endo et al. (2022) recently identified gene networks correlated with astrocyte morphology, including Alzheimer's disease (AD) risk genes. Interestingly, those genes were down‐regulated in APP/PSIdE9 mice that displayed reduced astrocyte morphology. It may be interesting to determine whether and how those astrocyte morphology–related genes are altered in the different clusters we identified in our Tau22 mice.

The persistence of the cluster with complex morphology in Tau22 mice is intriguing. There is some evidence of plasticity of perivascular astrocytes in vivo (Bardehle et al. 2013) or in neurotrauma, stroke, or neurodegeneration with hypertrophy of reactive astrocytes (Wilhelmsson et al. 2006). Following experimental ablation of a single astrocyte, astrocytes in the vicinity extend their processes to maintain vascular coverage. In 12‐month‐old mice, this response is delayed (Mills 3rd et al. 2022). In physiological conditions, the level of enrichment of the housing environment and learning can also induce morphological complexity of astrocytes in the hippocampus of young and old rats (Sampedro‐Piquero et al. 2014) and mice (Diniz et al. 2016). Hence, it is tempting to interpret the resilience of this complex astrocytic cluster as a mechanism to compensate for the simplification of the predominant cluster and maintain some level of homeostatic functions. Alternatively, these changes may be associated with a de novo gain of function. Elevated astrocytic and microglial phagocytosis of synapses laden with oligomeric tau was observed in cognitively impaired ad patients (Tzioras et al. 2023) but not in resilient ones (Taddei et al. 2023), suggesting that this synaptic pruning may be aberrant and at the root of cognitive decline. It is also possible that astrocytes change their morphology in order to contribute to the removal of debris or dead cells, along with microglia (Damisah et al. 2020) or to maintain tissue structure after cell loss. Although technically challenging, the question of the relationship between astrocyte shape and function could be addressed by combining spatial transcriptomics, 3D reconstruction, and functional assessment. It is likely, however, that changes in the physical contacts between astrocyte terminals and synapses, the vascular bed, and the terminals of other astrocytes contribute to astrocytic homeostatic dysfunctions in glutamate uptake and release, to name but one example.

**FIGURE 8:**
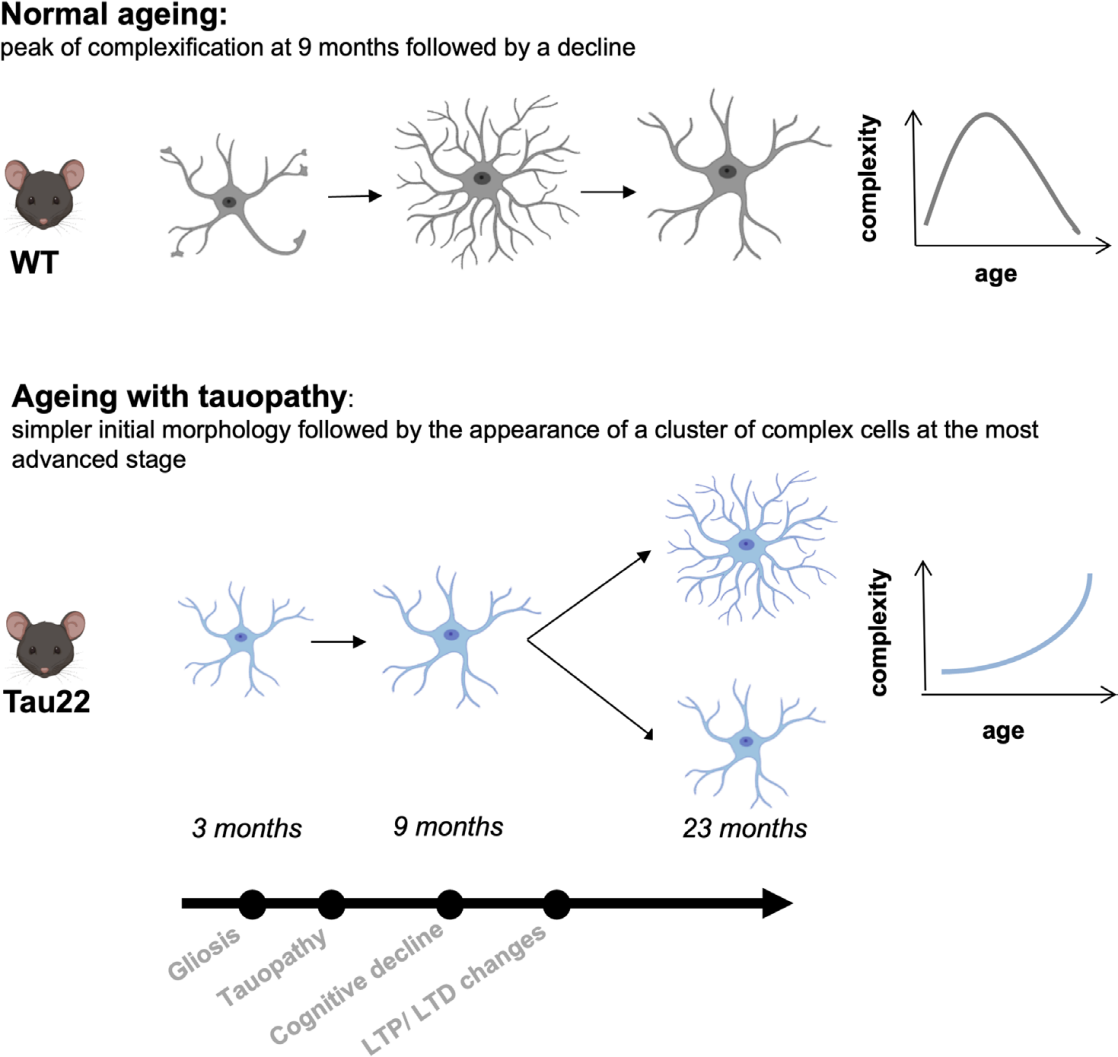
Hypothetical model showing the distinct trajectories of astrocytic morphological changes with age in WT and Tau22 mice. In WT mice, the complexity of morphology followed a bell‐shaped curve, reaching a peak at the adult age, followed by a significant atrophy at an advanced age. In contrast, the morphology of Tau22 astrocytes remained fairly simple throughout their lifespan, with the emergence of a small fraction of complex cells at an advanced age and as phenotypic changes evolves. Created with Biorender.

In conclusion, we have developed a pipeline to analyze the distribution of morphological subtypes of astrocytes in a mouse model of tauopathy at different stages of the disease. Our data show that as hippocampal astrocytes are progressively exposed to pathological tau, their morphological changes follow a different pathway from that of normal aging. While the majority of hippocampal astrocytes tend to simplify their shape, a non‐negligible fraction becomes more ramified and complex (Figure 8). Future studies will unravel the significance of such changes.

## Author Contributions

K.C., E.A., and G.B. designed the project, supervised the data analysis, and wrote the manuscript. E.A., T.V.S., M.R.P., M.G., G.A., J.M., C.Jo., C.Ja., A.S.H., F.P., D.P., and S.B. performed the experiments and analyzed the results. M.C.G., A.L., E.F., A.L.B., D.B., L.B., and A.‐P.B. contributed intellectual content and critical manuscript review.

## Conflicts of Interest

The authors declare no conflicts of interest.

## Supporting information


Data S1.

Data S2.

## Data Availability

Detailed datasets may be made available from the corresponding author upon request.
