# Supplementary material for: Hippocampal Astrocyte Morphology Follows an Unexpected Trajectory With Age in a Transgenic Rodent Model of Tauopathy
Source: Glia. 2025 Mar 22;73(7):1502–19. doi: 10.1002/glia.70019 (PMC12121468; doi:10.1002/glia.70019)
Supplement: Supplementary file 1 — Data S1. [file GLIA-73-1502-s001.docx]

**Supplementary materials**

**Title:** Hippocampal astrocyte morphology follows an unexpected trajectory with age in a transgenic rodent model of tauopathy

**Short running title**: Tauopathy affects astrocytic morphology

**Authors:** Emma Augustin^1^, Tatiana Vinasco-Sandoval^2^, Miriam Riquelme-Perez^1^, Damien Plassard^5^, Mylène Gaudin^1^, Gwenaëlle Aurégan^1^, Julien Mitja^1^, Sueva Bernier^1^, Charlène Joséphine^1^, Fanny Petit^1^, Caroline Jan^1,9^, Anne-Sophie Hérard^1^, Marie-Claude Gaillard^1^, Agathe Launay^3,4^, Emilie Faivre^3,4^, Luc Buée^3,4^, Anne-Laurence Boutillier^6,7,8^, David Blum^3,4^, Alexis Bemelmans^1^, Gilles Bonvento^1,9^, Karine Cambon^1^

**Affiliations :**

**1** Université Paris-Saclay, CEA, CNRS, MIRCen, Laboratoire des Maladies Neurodegeneratives, Fontenay-aux-Roses, France.

**2** CEA, CNRS, DRF, IBFJ, IRCM, Laboratoire de génomique et radiobiologie de la kératinopoeièse, 91 000, Evry, France

**3** Université de Lille, Inserm, CHU Lille, UMR-S1172 Lille Neuroscience & Cognition (LilNCog), Lille, France.

**4** Alzheimer and Tauopathies, LabEx DISTALZ, France.

**5** GenomEast Platform, Institut de Génétique et de Biologie Moléculaire et Cellulaire (IGBMC), CNRS UMR 7104, INSERM U1258, Université de Strasbourg, Illkirch, France

**6** Laboratoire de Neurosciences Cognitives et Adaptatives (LNCA), Strasbourg, France.

**7** Centre National de la Recherche Scientifique (CNRS, UMR 7364), Strasbourg, France.

**8** University of Strasbourg, Strasbourg, France

**9** present address: Université Paris-Saclay, CNRS, Institut des Neurosciences Paris-Saclay, Saclay, France.

**
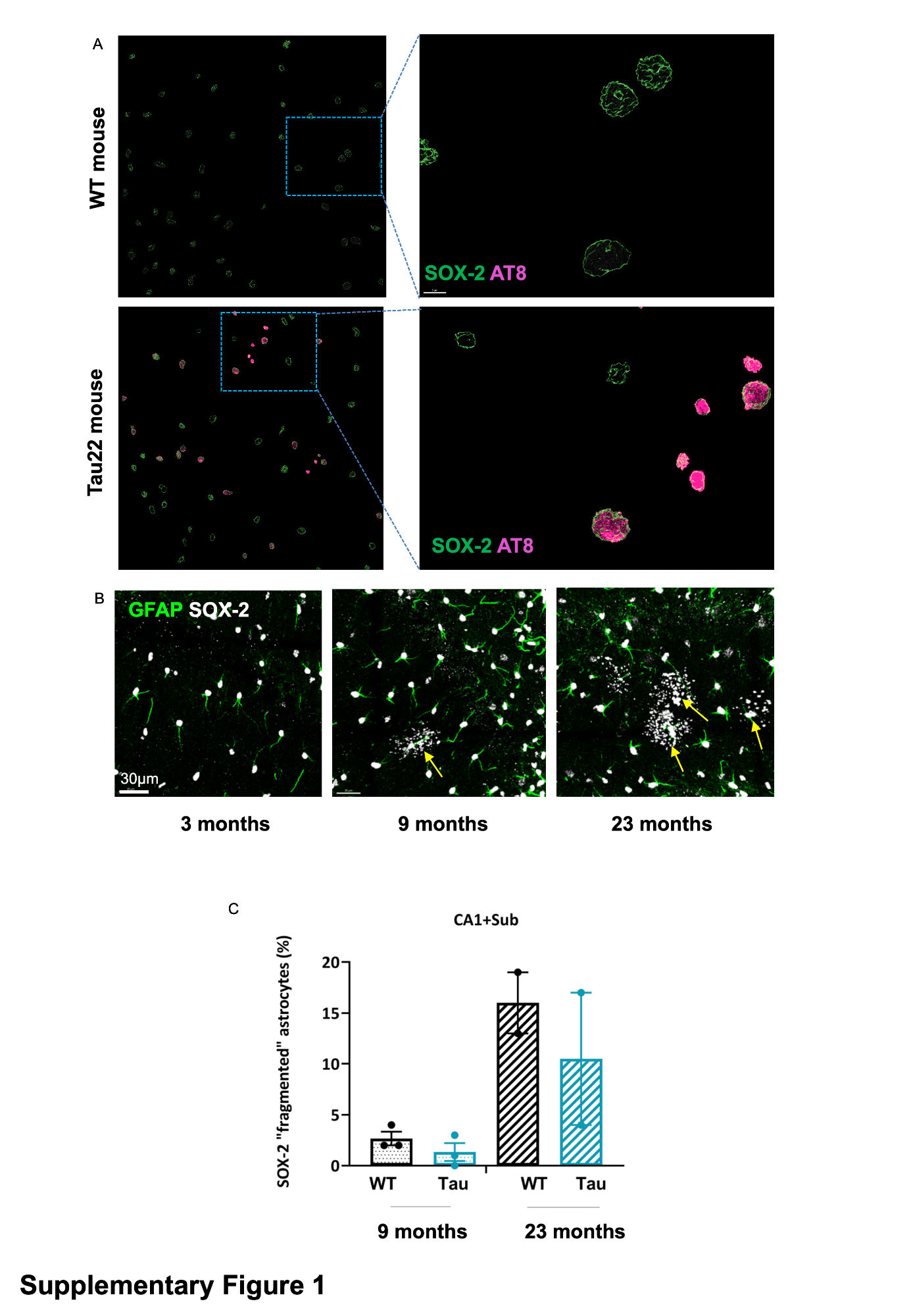
 Supplementary Figure 1** *(A) 3D- displays of AT8^-^/SOX-2^+^ cells (in green) and AT8^+^/SOX-2^+^ cells (in pink), as used for the calculation of the percentage of AT8^+^/SOX-2^+^ cells in the hippocampus. (B) Representative image of SOX-2/GFAP immunofluorescent staining performed on hippocampal sections of 3-, 9-and 23-month-old Tau22 mice. With age, more astrocytes displayed clasmatodendrosis, detected by the abnormal relocation of SOX-2 staining from the nucleus to their processes, suggesting a fragmentation of their cytoskeleton. (C) Quantification of the % of astrocytes displaying a clasmatodendrosis phenotype within a whole hippocampal section. None were detected in 3-month-old mice. All data is presented as mean ±SEM, with n= 2-3 mice per genotype. CA1, Cornu Ammonis 1; Sub, subiculum.*

**
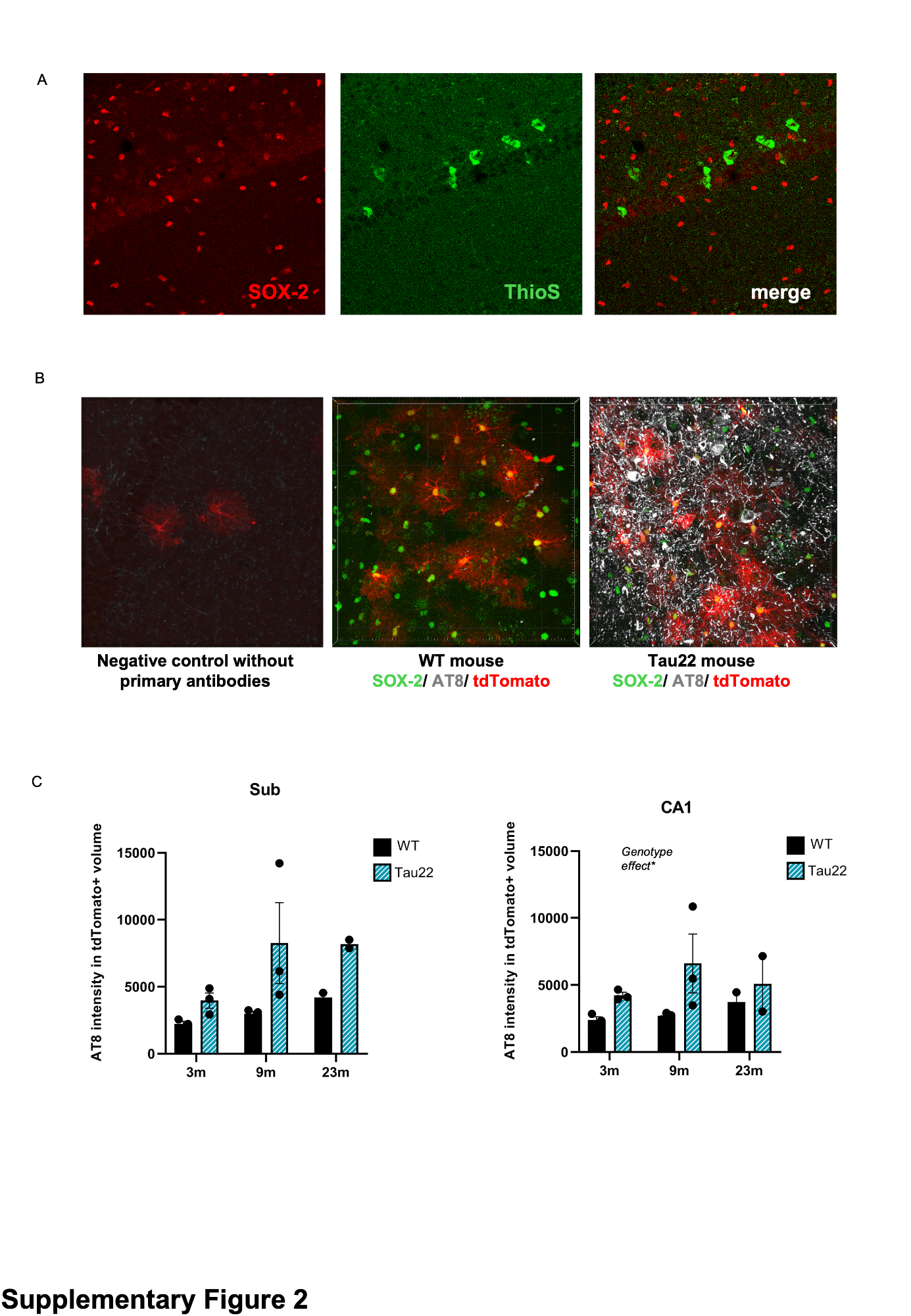
 Supplementary Figure 2.** (A) *Representative image of SOX-2/Thio S immunofluorescent staining performed on hippocampal sections of 18-month-old Tau22 mice, showing that mature tau aggregates accumulate in pyramidal CA1 neurons only, and not in astrocyte. (B) Representative images of AT8/SOX-2/tdTomato immunofluorescent staining in WT and Tau22 mice. (C) Quantification of the AT8 immunostaining intensity within the tdTomato-positive volume in a 40µm-thick section of the hippocampus in the CA1 region and the subiculum for the 3 age groups in WT and Tau22 mice (intensity of voxels per µm^3^). Data plotted are the values of AT8 sum intensity within the whole tdTomato^+^ volume for individual mice (dots), and mean± SEM for each group. Data were analyzed with two-way ANOVA with genotype and age as factors. *p<0.05. CA1, Cornu Ammonis; Sub, subiculum.*

**
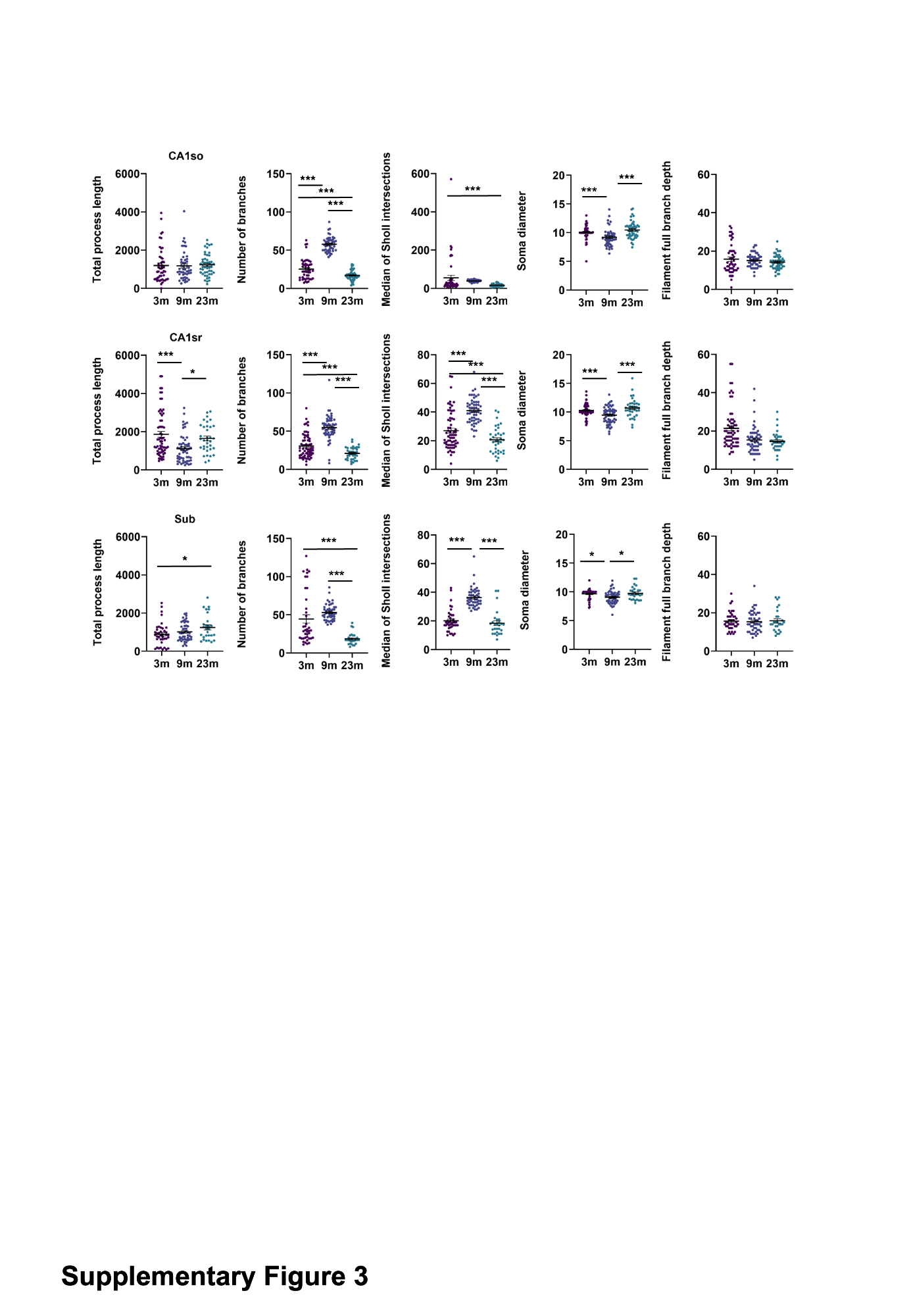
 Supplementary Figure 3.** *Astrocytes morphology acquires complexity during adulthood before simplifying at old age in WT mice. A selection of morphological features from 3D-reconstructed astrocytes from WT mice are shown. They were compared between the different ages (3-, 9- and 23-month-old). Data plotted are individual astrocyte values (dots) from 2 to 3 mice /group, and mean± SEM for each group. Data were analysed with one way ANOVA. *p<0.05, ** p<0.01, *** p<0.001. CA1, Cornu Ammonis; CA1so, CA1 stratum oriens; CA1sr, CA1 stratum radiatum ; Sub, subiculum. Total process length and soma diameter are expressed in microns.*

**
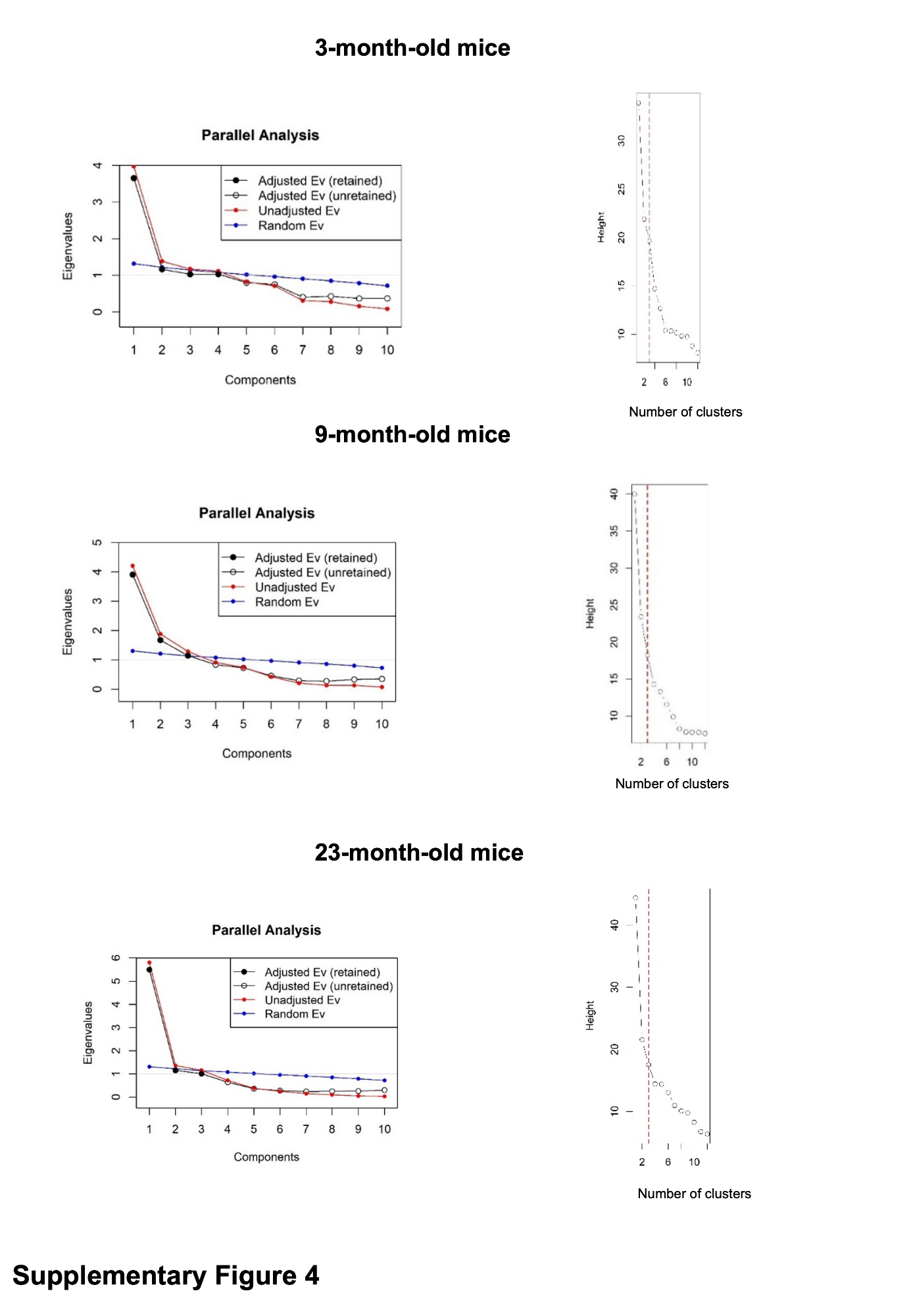
 Supplementary Figure 4** *(Left) Horn’s parallel analysis was used to determine the appropriate number of principal components. The first 3 PCs showed eigenvalues greater than 1 and were retained. (Right) A scree plot of dendrogram height allows the selection of the appropriate number of clusters to cut the tree. Here, in these datasets, the optimal number of clusters is three.*

**
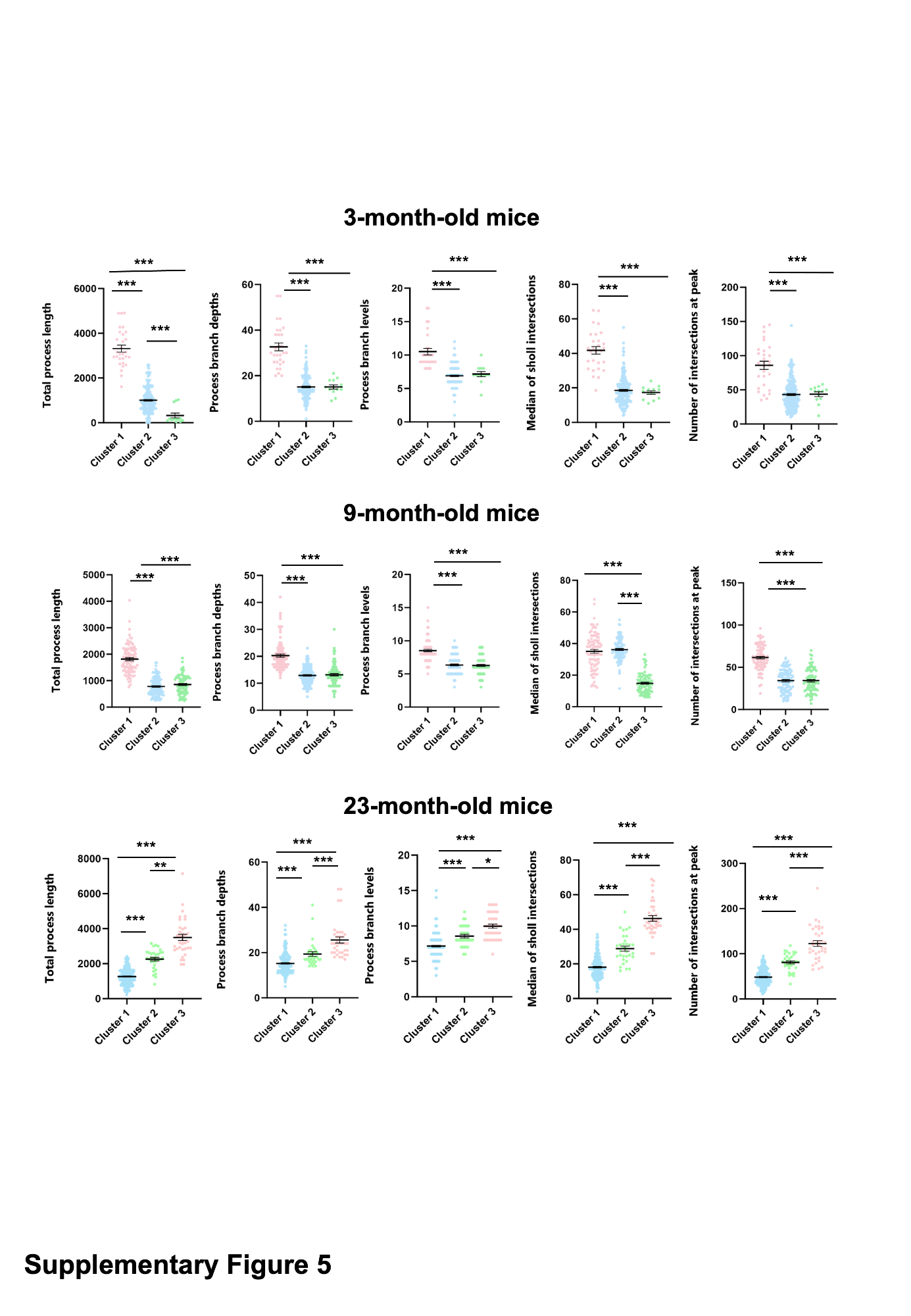
Supplementary Figure 5 Morphological features of identified astrocyte clusters.**

*Selection of morphological features for astrocytes in clusters 1, 2 and 3 for 3- and 23-month-old mice. Data plotted are individual astrocyte values (dots) from 2 to 3 mice /group from all subregions together, and mean± SEM for each group in black. Data were analyzed with Kruskall Wallis. *p<0.05, ** p<0.01, *** p<0.001.*
