# Supplementary material for: Hippocampal Astrocyte Morphology Follows an Unexpected Trajectory With Age in a Transgenic Rodent Model of Tauopathy
Source: Glia. 2025 Mar 22;73(7):1502–19. doi: 10.1002/glia.70019 (PMC12121468; doi:10.1002/glia.70019)
Supplement: Supplementary file 2 — Data S2. [file GLIA-73-1502-s002.docx]

| **Morphological feature** | **Definition** |
| --- | --- |
| Total process length | Sum of the length of all tdTomato-positive branches |
| Total number of branches | Sum of all tdTomato-positive branches |
| Total number of branching points | Number of points of ramification between two branches |
| Median of Sholl intersections | Median number of intersections of the Sholl profile |
| Process branch level | Highest value of branch level for the dendritic graph |
| Process branch depth | Number of branch points in the shortest path from the beginning point |
| Total number of primary branches | Number of branches emanating from the soma |
| Number of intersections at peak | Number of intersections at the peak of the Sholl profile |
| Ramification index | Equal to the ratio of the number of intersections at the peak of the Sholl profile/ total number of primary branches |
| Soma diameter | Length of the soma measured in the X Y plane |

|  |  |  |  |  |  |  |  |  |  |  |  |  |
| --- | --- | --- | --- | --- | --- | --- | --- | --- | --- | --- | --- | --- |
| **Age** | **Sub-region** | **Genotype** | **Total process length** | **Total number of branches** | **Total number of branching points** | **Median of sholl intersections** | **Process branch levels** | **Process branch depths** | **Total number of primary branches** | **Number of intersections at peak** | **Ramification index** | **Soma diameter** |
|  |  |  | **mean +/- SEM** | **mean +/- SEM** | **mean +/- SEM** | **mean +/- SEM** | **mean +/- SEM** | **mean +/- SEM** | **mean +/- SEM** | **mean +/- SEM** | **mean +/- SEM** | **mean +/- SEM** |
| **3-month-old** | **Stratum Oriens** | **WT** | 1198 +/- 128 | 25,28+/-1,92 | 120+/-15,57 | 55,21+/-13,64 | 7,14+/-0,29 | 15,80+/-1,11 | 9,35+/-0,4 | 51,98+/-4,65 | 5,74+/-0,53 | 10,05+/-0,17 |
|  |  | **Tau22** | 1004 +/- 147,5 | 29,39+/-4,35 | 96,88+/-17,13 | 17,82+/-1,83 | 7,03+/-0,31 | 16,24+/-1,08 | 8,39+/-0,59 | 48,26+/-3,71 | 6,25+/-0,52 | 9,22+/-0,24 |
|  |  | **Nested ANOVA** | F=1,65 ; P> 0,05 | F=3,96 p>0,05 | F=2,56 p>0,05 | F=13,11 p<0,05 | F=0,014 p>0,05 | F=0,43 p>0,05 | F=6,18 p<0,05 | F=0,3 p>0,05 | F=0,0057 p>0,05 | F=11,17 p<0,05 |
|  | **Stratum Radiatum** | **WT** | 1864 +/- 149 | 30,55+/-1,94 | 183,4+/-17,11 | 27,04+/-1,67 | 8,29+/-/0,32 | 21,44+/-1,36 | 7,86+/-0,28 | 57,24+/-3,24 | 8,01+/-0,67 | 10,28+/-0,13 |
|  |  | **Tau22** | 1151 +/- 140 | 21,43+/-2 | 111,6+/-14,24 | 19,41+/-1,63 | 7,17+/-0,29 | 17,09+/-1,1 | 8,26+/-0,54 | 37,03+/-2,07 | 4,86+/-0,32 | 10,52+/-0,31 |
|  |  | **Nested ANOVA** | F=13,7 ; P<0,05 | F=9,69 p<0,05 | F=13,9 p<0,05 | F=9,6 p<0,05 | F=5,05 p<0,05 | F=5,43 p<0,05 | F=0,052 p>0,05 | F=16,8 p<0,05 | F=8,05 p<0,05 | F=4,97 p<00,05 |
|  | **Subiculum** | **WT** | 893 +/-91 | 44,60+5,38 | 274,12+/-55,08 | 20,09+/-1,19 | 7,25+/-0,19 | 15,7+/-0,73 | 8,28+/-0,43 | 41,5+/-3,29 | 5,15+/-0,36 | 9,65+/-0,14 |
|  |  | **Tau22** | 826 +/-51,7 | 17,41+/-1,14 | 74,97+/-6,14 | 16,06+/-0,81 | 6,32+/-0,23 | 13,15+/-0,59 | 8,09+/-0,47 | 44+/-4,65 | 5,56+/-0,53 | 9,68+/-0,18 |
|  |  | **Nested ANOVA** | F= 0,23; P>0,05 | F=84,13 p<0,05 | F=40,9 p<0,05 | F=6,1 p<0,05 | F=6,56 p<0,05 | F=7,66 p<0,05 | F=1,17 p>0,05 | F=0,02 p>0,05 | F=1,55 p>0,05 | F=0,082 p>0,05 |
| **9-month-old** | **Stratum Oriens** | **WT** | 1181+/-107 | 57,83+/-1,4 | 96,94+/-10 | 38,67+/-0,8 | 7,60+/-0,3 | 15,15+/-0,5 | 6,02+/-0,3 | 46,23+/-2,8 | 8,76+/-0,7 | 9,18+/-0,2 |
|  |  | **Tau22** | 1006+/-79 | 18+/-1,3 | 83,5+/-7,7 | 21+/-1,6 | 6,7+/-0,2 | 16,1+/-0,9 | 6,8+/-0,3 | 38,4+/-2,6 | 6+/-0,4 | 10,1+/-0,2 |
|  |  | **Nested ANOVA** | F= 1,6; P> 0,05 | F=318 p<0,05 | F=1,49 p>0,05 | F=88,5 p<0,05 | F=2,33 p>0,05 | F=0,81 p>0,05 | F=0,37 p>0,05 | F=2,8 p>0,05 | F=4,36 p<0,05 | F=9,82 p<0,05 |
|  | **Stratum Radiatum** | **WT** | 1114+/-98 | 54,71+/-2,1 | 88,93+/-8 | 40,96+/-1,1 | 6,75+/-0,3 | 15,35+/-0,9 | 6,29+/-0,3 | 39,84+/-2,4 | 7,31+/-0,7 | 9,45+/-0,2 |
|  |  | **Tau22** | 1271+/-98 | 20,85+/-1,7 | 100,05+/-8,8 | 17,71+/-1,2 | 7,08+/-0,3 | 15,42+/-0,8 | 6,73+/-0,3 | 44,19+/-2,9 | 7,11+/-0,7 | 10,62+/-0,3 |
|  |  | **Nested ANOVA** | F= 2,65;P> 0,05 | F=136,25 p<0,05 | F=2,05 p >0,05 | F=193 p<0,05 | F=1,94 p>0,05 | F=0,087 p>0,05 | F=0,92 p>0,05 | F=3,13 p>0,05 | F=0,13 p>0,05 | F=9,37 p<0,05 |
|  | **Subiculum** | **WT** | 1013+/-68 | 53,19+/-1,5 | 87,83+/-6,4 | 36,32+/-1 | 7,19+/-0,3 | 15,36+/-0,8 | 6,6+/-0,3 | 41,4+/-2,5 | 6,71+/-0,5 | 9,08+/-0,2 |
|  |  | **Tau22** | 1220+/-74 | 20,23+/-1,3 | 102,93+/-7,7 | 17,88+/-0,9 | 6,83+/-0,2 | 14,76+/-0,7 | 7,83+/-0,4 | 49,1+/-2,9 | 6,64+/-0,5 | 10,22+/-0,3 |
|  |  | **Nested ANOVA** | F= 5,67;P<0,05 | F=246,3 p<0,05 | F=3,23 p>0,05 | F=191 p<0,05 | F=1,09 p>0,05 | F=0,26 p>0,05 | F=7,75 p<0,05 | F=5,81 p<0,05 | F=0,006 p>0,05 | F=15,82 p<0,05 |
| **23-month-old** | **Stratum Oriens** | **WT** | 1233+/-80,7 | 17,11+/-0,9 | 88+/-6,6 | 16,5+/-1 | 6,82+/-0,2 | 14,22+/-0,5 | 5,92+/-0,3 | 47,84+/-3,1 | 8,46+/-0,5 | 10,48+/-0,2 |
|  |  | **Tau22** | 1566+/-90,8 | 25,31+/-1,6 | 147,58+/-11,4 | 22,95+/-1,3 | 7,87+/-0,2 | 17,78+/-0,7 | 8,03+/-0,4 | 60,24+/-3,5 | 8,08+/-0,5 | 10+/-0,2 |
|  |  | **Nested ANOVA** | F=6,45;P<0,05 | F=21,28 p<0,05 | F=25 p<0,05 | F=17,5 p<0,05 | F=9,03 p<0,05 | F=14,98 p<0,05 | F=9,76 p<0,05 | F=7,72 p<0,05 | F=0,228 p>0,05 | F=6,65 p>0,05 |
|  | **Stratum Radiatum** | **WT** | 1646+/-124 | 21,11+1,4 | 115,57+/-10,7 | 20,6+/-1,5 | 6,77+/-0,3 | 14,6+/-0,8 | 6,97+/-0,3 | 57,23+/-4,1 | 8,5+/-0,7 | 10,71+/-0,3 |
|  |  | **Tau22** | 2237+/-176 | 30,57+/-1,9 | 197,59+/-18,2 | 29+/-1,9 | 8,06+/-0,3 | 18,32+/-0,7 | 8,78+/-0,4 | 77,43+/-5,7 | 9,33+/-0,7 | 10,36+/-0,2 |
|  |  | **Nested ANOVA** | F= 54,24;P<0,05 | F=9,11 p<0,05 | F=8,27 p<0,05 | F=7,47 p<0,05 | F=8,47 p<0,05 | F=10,69 p<0,05 | F=5,52 p<0,05 | F=3,74 p>0,05 | F=0,117 p>0,05 | F=1,24 p>0,05 |
|  | **Subiculum** | **WT** | 1247+/-127 | 18,52+/-1,5 | 104,07+/-13,5 | 18,37+/-1,7 | 7,63+/-0,3 | 15,81+/-1,1 | 6,96+/-0,4 | 48,56+/-4,9 | 7,2+/-0,7 | 9,77+/-0,2 |
|  |  | **Tau22** | 1842+/-130 | 30,47+/-2,4 | 177,42+/-14 | 28+/-2,2 | 8,91+/-0,3 | 21,24+/-1,7 | 8,79+/-0,6 | 72,64+/-5,4 | 8,81+/-0,7 | 10,26+/-0,2 |
|  |  | **Nested ANOVA** | F= 13,86;P<0,05 | F=22,5 p<0,05 | F=27,6 p<0,05 | F=22,34 p<0,05 | F=11,3 p<0,05 | F=13,15 p<0,05 | F=5,67 p<0,05 | F=12,43 p<0,05 | F=3,49 p>0,05 | F=2,95 p>0,05 |
